# Recent Advances in Bioconjugation of Aromatic Amino Acid Residues by a Reactivity‐Guided Approach

**DOI:** 10.1002/tcr.202500215

**Published:** 2025-11-26

**Authors:** Bruno M. da S. Santos, Lívia C. R. M. da Frota, Thais G. Silva, Fernanda G. Finelli

**Affiliations:** ^1^ Instituto de Pesquisas de Produtos Naturais Walter Mors Universidade Federal do Rio de Janeiro Rio de Janeiro Brazil; ^2^ Instituto de Química Universidade Federal do Rio Grande do Sul Rio Grande do Sul Brazil

**Keywords:** aromatic amino acids, bioconjugation, chemical biology, radical reactions, transition metal catalysis

## Abstract

The bioconjugation of aromatic amino acids has emerged as a powerful strategy in chemical biology, drug discovery, and biomolecular research. Beyond the classical targeting of cysteine and lysine, aromatic amino acids residues offer higher selectivity owing to their lower abundance and critical roles in intermolecular interactions. Current synthetic approaches include substitution reactions, addition reactions, free‐radical reactions, metal‐catalyzed transformations, and biocatalytic approaches, enabling precise and versatile modifications in cells, tissues, and at the proteome level. In recent years, transition‐metal catalysis and radical processes have dominated the field, with particular emphasis on tyrosine and tryptophan. This review provides a critical analysis of advances from the past 3 years, categorizing methodologies by reaction mechanism and highlighting how the intrinsic reactivity of aromatic amino acids can be harnessed for site‐selective functionalization, ultimately expanding the accessible chemical space across all these residues.

## Graphical abstract

This review highlights recent advances in the bioconjugation of aromatic amino acids residues, focusing on strategies that leverage their inherent chemical reactivity to enable precise and versatile modifications of biomacromolecules, illustrating relevant applications.

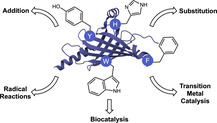

## 1 Introduction

The utility of protein and peptide bioconjugates continues to expand, finding critical roles in biomedicine [1], molecular diagnostics [2], and basic biology research [3]. These entities enable the creation of targeted drug delivery systems [4, 5], fluorescent probes for in vivo and in vitro visualization [6], and various derivatizations for studying biomolecular dynamics and interactions [7, 8].

Moving beyond classical genetic engineering methods that incorporate noncanonical amino acids into the protein sequence, recent approaches prioritize the direct chemical modification of wild‐type proteins post‐translationally [9]. The inherent nucleophilicity of cysteine and lysine residues has established them as primary targets for conventional bioconjugation strategies [10, 11]. However, these methods usually face obstacles, such as the need of reduction of native disulfide bonds for cysteine conjugation and the heterogeneity and inactivation resulting from the overlabeling of lysine residues. In this sense, aromatic amino acids present an attractive alternative. Their lower natural abundance and reduced reactivity offer a pathway to achieve more precise and controlled labeling [12]. They also participate in key biomolecular interactions, such as *π*–*π*, cation−*π*, and CH−*π*, which are responsible for dominant forces in protein folding, structure, and functional interactions at active sites [13].

Recent years have seen a growing number of innovative methodologies aimed at functionalizing these residues, addressing the challenge of balancing reactivity with selectivity under biocompatible conditions. These developments demonstrate that, despite the complexity of biomacromolecules, their modification can be approached by applying fundamental concepts of synthetic organic chemistry, understanding underlying reactivity patterns as with small molecules.

While recent reviews cover transformations targeting specific aromatic residues [14, 15, 16, 17, 18, 19, 20] or specific modification methodologies [21, 22, 23, 24, 25, 26], a unified discussion through the lens of inherent chemical reactivity would provide a valuable framework for the field, especially given its rapid pace of growth.

This review is organized according to the fundamental transformation undergone by amino acid side chains. From this perspective, classical reactions are categorized based on whether the side chain participates in an electrophilic aromatic substitution or an addition reaction. For clarity, reactions targeting the heteroatoms of the side chain are discussed separately. Finally, distinct sections are devoted to transformations enabled by specific catalytic strategies, including biocatalysis, transition‐metal catalysis, and radical processes. This review specifically highlights the advances achieved over the past 3 years.

## 2 Electrophilic Aromatic Substitution Reactions (EAS)

Electrophilic aromatic substitution reactions represent the most classic reactivity pattern of aromatic compounds and constitute a reliable and versatile toolbox for aromatic ring functionalization. During the last 3 years, they were explored to enable the formation of C–C, C–S, C–N, and C–Halogen bonds in peptides and proteins, leading to valuable applications in medicinal chemistry and the development of fluorescent probes for cancer cell detection research. Tryptophan (Trp) and tyrosine (Tyr) were the most widely targeted residues for these reactions, and excellent selectivity for either residue could be achieved through careful control of reaction conditions.

**SCHEME 1:**
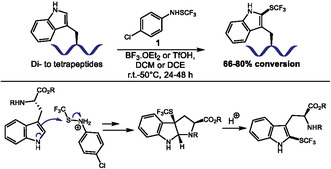
Trifluoromethylthiolation of Trp residues in peptides and proposed intermediates.

The sulfenylation, especially the trifluoromethylthiolation of aromatic amino acids, can significantly impact their biophysical properties, but reactions usually struggle from low yields for Trp derivatization and do not achieve Tyr functionalization. In 2012, Billard, Langlois, and coworkers reported the use of *p*‐toluenesulfonic acid‐activated trifluoromethanesulfenamide reagents to promote the efficient trifluoromethylthiolation of Indoles and electron‐rich arenes, but their methodology achieved low yields for Trp [27]. Iskra group later demonstrated the higher stability of the *p*‐chloro‐trifluoromethanesulfenamide derivative (**1**) [28]. Building on these findings, in 2023, Brigaud, Iskra, Chaume, and coworkers published the synthesis of trifluoromethylthiolated aromatic amino acids using **1** (Scheme 1) [29]. The authors show that Trp reacts smoothly using BF_3_·OEt_2_ as activator, while Tyr derivatives require both stronger triflic acid (TfOH) activation and protection of the amino group to proceed. The method was also applied for late‐stage regioselective modification of Trp residues in peptides. The scope encompassed fluorenylmethoxycarbonyl‐protected (Fmoc‐protected) and *N*‐unprotected di‐, tri‐ and tetrapeptides, obtained in 66–80% yield across 8 examples. The authors showed high Trp selectivity over Tyr residues, while phenylalanine (Phe) residues remained untouched. Selectivity against Histidine (His) and other nucleophilic side chains was not evaluated. The mechanism proceeds via electrophilic attack, facilitated by acid activation of sulfenamide. For Trp, initial cyclization to CF_3_S‐pyrroloindoline intermediates occurs, followed by acid‐mediated ring‐opening to the final product.

The authors also demonstrated that incorporation of CF_3_S‐Tyr/Trp derivatives into endomorphin‐1 (EM‐1) via solid‐phase synthesis, enhanced chromatographic hydrophobicity indexes by outstanding 8–12 units, the highest shift measured using this method. CF_3_S group also lowers the pK_a_ of Tyramine's phenol by 1.8 units. The increase in local hydrophobicity and modulation of the pKa of near groups can facilitate rational design of bioactive peptides with improved membrane permeability and acidities for medicinal chemistry.

**SCHEME 2:**
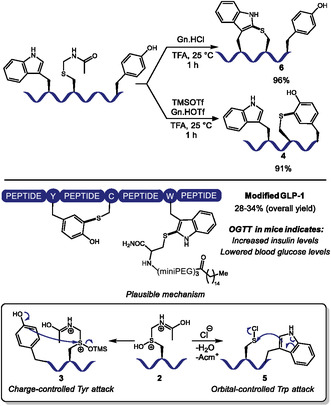
Chemoselective sulfenylation of Trp or Tyr residues. One‐pot sequential stapling and lipidation of GLP‐1 derivative with significant biological relevance. Plausible mechanism that accounts for exchangeable condition‐dependant chemoselectivity.

A few years ago, building on Yajima's observation of how *S‐para*‐methoxybenzyl cysteine sulfoxide (Cys(MBzl(O)) was converted into *S‐para*‐methoxyphenyl cysteine under acidic conditions in the presence of anisole [30], Otaka and coworkers presented the use of this oxidized cysteine derivative with guanidine hydrochloride (Gn·HCl) to achieve peptide cyclization through the sulfenylation of Trp residues [31]. In this report, reaction conditions were not suitable for Tyr sulfenylation. In 2023, the group advanced to an acid‐controlled strategy for chemoselective C–H sulfenylation of Tyr or Trp in peptides (Scheme 2) [32]. Using *S*‐acetamidomethyl cysteine sulfoxide **2** (Cys(Acm)(O)) as electrophile, selective Tyr‐sulfenylation was achieved with trimethylsilyl trifluoromethanesulfonate (TMSOTf) and guanidinium triflate (Gn‐HOTf) in trifluoroacetic acid (TFA) or hexafluoroisopropanol (HFIP), while Trp‐sulfenylation was achieved with Gn·HCl in TFA. This interesting control over selectivity is ascribed to a change in the reactive intermediates: a dicationic intermediate **3** is formed from Cys(Acm)(O) and TMSOTf, which favors the charge‐controlled Tyr electrophilic attack leading to Cys‐Tyr adducts **4**. On the other hand, in the presence of a chloride source, *S*‐chlorocysteine **5** is formed, which favors a more orbital‐controlled Trp attack, leading to Cys‐Trp adducts **6**. Reaction scope involved model peptides to confirm selectivity in the presence of His, Phe, lysine (Lys), serine (Ser), and methionine (Met) residues. Electrostatic repulsion to terminal ammonium ion probably hindered N‐terminal Trp modification, which was unsuccessful.

The method enabled one‐pot stapling and subsequent lipidation of glucagon‐like peptide‐1 (GLP‐1) analogs. Treatment of wild‐type mice with native GLP‐1 and the doubly modified peptides showed enhanced hypoglycemic activity: oral glucose tolerance tests (OGTT) revealed significantly lower blood glucose levels in the animals treated with the modified peptides, demonstrating utility for diabetes treatment.

**SCHEME 3:**
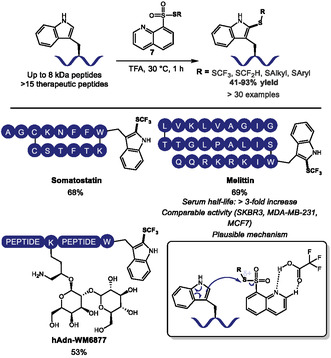
*C*2 sulfenylation of tryptophan‐containing peptides. Selected examples, biological application and proposed mechanism.

In 2024, Li, Liu, and coworkers developed a method for *C*2 sulfenylation of tryptophan residues in unprotected peptides and peptide drugs using 8‐quinoline thiosulfonates (**7**) as sulfur donors and TFA as both solvent and activator (Scheme 3) [33]. Their reaction tolerated high substrate concentrations and hydrophobic sequences, installing diverse thioether groups (fluoroalkyl, alkyl, aryl) in 41%–93% yields, including cyclic, linear peptides and glycopeptides without disrupting glycosylation. The thiosulfonate scope also introduced synthetically useful functional handles, such as carboxylic acids, alcohols, azides, and alkynes, for further modification. Mechanistic studies revealed TFA's dual role: activating the thiosulfonate via hydrogen‐bonding, thus enhancing sulfur electrophilicity; and protonating competing nucleophiles such as Lys, ensuring Trp selectivity, even over other aromatic residues like Tyr and His.

The authors also showed the applicability of the methodology through the increase in serum stability of melittin, a 26 amino acid peptide found in honeybee venom that has remarked activities against several cancer types. The synthesized *C*2‐SCF_3_ and *C2*‐SCF_2_H analogs retained or slightly improved their activity against different breast cancer cell lines and remarkably extended serum half‐life from 8 h to more than 24 h. This increased stability is ascribed to the inhibition of peptidase cleavage which can also increase the pharmacokinetic properties and boost the therapeutic uses of these modified peptides.

**SCHEME 4:**
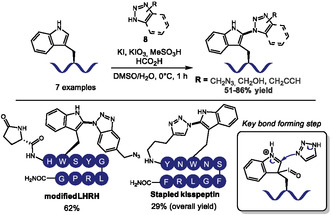
*C–N* coupling of triazoles and tryptophan‐containing peptides. Selected examples and key mechanistic step.

The formation of C—N bonds is also of fundamental importance, given their stability under physiological conditions. Several nitrogen compounds can then be used as viable and biocompatible linkers or markers for bioconjugation. Iodine‐based oxidants like *N*‐iodosuccinimide have been previously used to promote the C–N coupling of Trp with azoles in protected peptides in organic solvent [34]. In 2023, Hanaya and coworkers proposed an iodine‐mediated C2–N coupling at tryptophan in unprotected polypeptides in aqueous based media (Scheme 4) [35]. Their method used in situ*‐*generated HIO_2_ from KIO_3_ and KI in acidic water/DMSO mixture to activate Trp toward the nucleophilic attack of azole groups like benzotriazole‐ and 1,2,3‐triazole derivatives (**8**) at the C2 position, with yields ranging from 51%–86%. Given the acidic media, more basic 1,2‐ and 1,3‐azoles were not suitable for the reaction. The scope included 7 biologically relevant peptides and modified triazoles with synthetically useful handles like azides and terminal alkynes. Selective Trp modification was always observed, which was ascribed to the strong acidic media preventing the reaction with other nucleophilic amino acids and Tyr. Formic acid was used to suppress methionine oxidation, although it was still present in minor proportions in some examples. Reactions with peptides containing N‐terminal Trp failed, probably due to electrostatic repulsion with the ammonium ion. The method enabled the peptide stapling of kisspeptin after reductive amination of Nterminus with an aldehyde equivalent triazole, yielding the macrocyclic analog in moderate yield.

**SCHEME 5:**
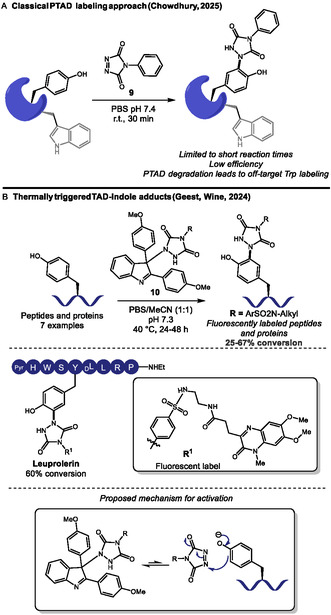
Tyrosine‐selective TAD‐functionalization. (A) Classical PTAD approaches. (B) Thermally triggered triazolinedione‐indole adducts.

Since Barbas and coworkers introduced phenyl triazolinediones (PTAD) reagents for aqueous ene‐type biorthogonal labeling of Tyr residues [36], they have been used as stable linkers to relevant handles [37] and to provide insights into protein structure [38], showcasing potential for probing protein interactions and proteomics. Chowdhury and coworkers employed PTAD (**9**) to modify surface‐exposed tyrosine residues under physiological conditions (Scheme 5A) [39]. They successfully labeled tyrosine in peptides, like neurotensin, and purified proteins, like bovine serum albumin (BSA), myoglobin, *β*‐casein, and carbonic anhydrase, with usually one or two labeled Tyr residues in each protein. They also tackled a complex protein mixture with HeLa cell lysates, tagging 31 proteins. In these complex mixtures, reaction efficiency drops due to steric hindrance from buried residues and potential deactivation of tyrosine through phosphorylation from metabolic cell activities. Also, as usually observed, hydrolysis of PTAD to phenyl isocyanate can lead to off‐target lysine and tryptophan labeling, which must be carefully avoided with short reaction times, ensuring Tyr selectivity.

In 2024, Geest, Winne, and coworkers significantly advanced this area by developing thermally triggered, blocked triazolinedione‐indole (**10**, TAD*) adducts [40]. TAD* reagents are bench‐stable and release active TAD selectively at 40°C, which can work as linker for several relevant functionalities like fluorescent labels. This controlled release enabled highly selective Tyr conjugation in concentrated pH 7.3 buffered media, rendering several labeled peptides like bivalirudin, leuprolerin, and neurotensin in 25%–60% yield (Scheme 5B). The methodology was also applied to proteins like insulin, lysozyme, and *α*‐lactalbumin with good chemo‐ and site‐selectivity, achieving single modification at 40%–67% conversion, although higher labeling ratios could be achieved in more forcing conditions. The authors also reported that reactions with myoglobin fail. Efficiency of the reaction sharply dropped in pure aqueous buffer, and c.a. 20% organic solvent like DMSO or CH_3_CN was required. After active TAD release, a classical electrophilic aromatic substitution reaction with Tyr proceeded, and the observed selectivity was ascribed to both the reaction pH, where Tyr population is partially deprotonated, and to the gradual and reversible TAD release, minimizing hydrolysis and suppressing slower off‐target tryptophan labeling, which was confirmed *via* competition studies. At lower pH 4, full protonation of Tyr residues slowed down their reaction, rendering a complete selectivity change toward Trp modification. The authors described that the electronic microenvironment of the Tyr residue is of outmost importance for selectivity, since their hydrogen‐bonding net can influence relative changes in pKa values and impact nucleophilicity.

**SCHEME 6:**
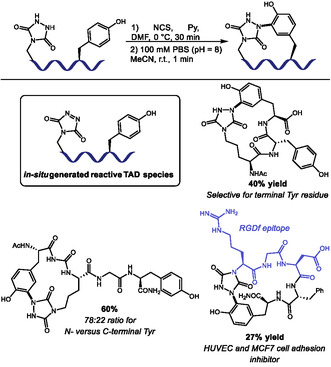
TAD‐promoted peptide macrocyclization.

In a recent work, Roberts and coworkers explored TAD labeling of Tyr residues to achieve a one‐pot, oxidation‐induced macrocyclization (Scheme 6) [41]. The method involves installing a urazole on the peptide, which is then oxidized with *N*‐chlorosuccinimide (NCS) to generate a reactive TAD moiety. Subsequent macrocyclization, triggered by dilution into a 3:2 MeCN/100 mM phosphate buffer at pH 8, yielded 23 Tyr‐linked cyclic peptides ranging from 3 to 11 residues, with yields of 10%–73%. The scope showed compatibility with histidine, lysine, serine, aspartic acid, and arginine. However, the presence of cysteine and tryptophan residues led to complex reaction mixtures due to oxidation of their side chains. The authors demonstrated high selectivity for terminal Tyr residues over internal ones, with a preference for N‐terminal Tyr, although the origin of this preference is unclear. The utility of the methodology was showcased by synthesizing two cyclic peptides containing the anti‐angiogenic RGDf epitope, which exhibited cell adhesion inhibition in MCF7 and human umbilical vein endothelial cells (HUVEC) comparable to the drug cilengitide, underscoring the method's potential for generating bioactive macrocycles.

**SCHEME 7:**
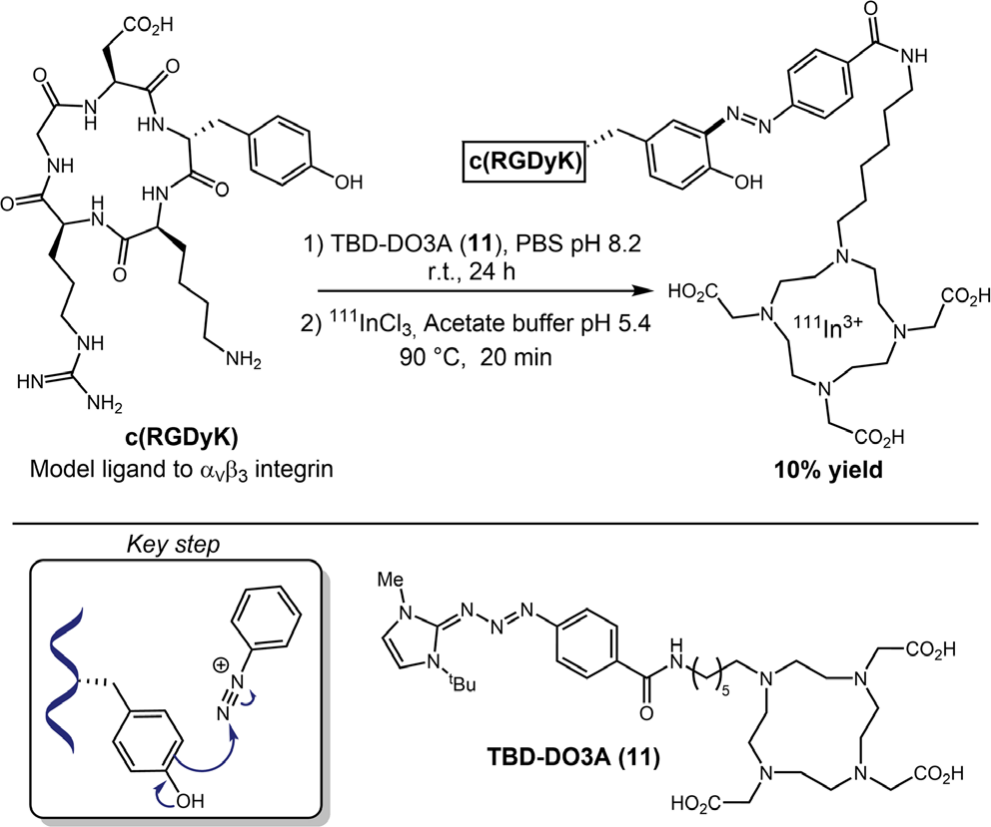
TBD‐DO3A as a radiolabeling handle for peptides, promoting selective tumor accumulation.

Selective azo coupling of diazonium salts to Tyr residues has been previously explored for different purposes like chemoproteomic and radiolabeling [42, 43]. After the pioneering work of Jewett and coworkers [44] on water‐soluble triazabutadienes (TBD) that can slowly release diazonium salts in reaction media under physiologically relevant pH, this strategy was employed, for example, to insert click handles for bioconjugation reactions to Tyr [45, 46]. Recently, Watanabe, Ono, and coworkers promoted a tyrosine‐specific radiolabeling by introducing TBD‐DO3A (**11**), a bifunctional reagent combining a TBD scaffold with DO3A chelator, which enabled conjugation under mild physiological conditions (Scheme 7) [47]. The methodology exploited TBD's ability to release aryl diazonium ions at physiological pH for azo coupling with tyrosine residues through electrophilic aromatic substitution. The authors promoted the modification of the cyclic peptide c(RGDyK) in 11% yield, followed by ^111^In radiolabeling. This peptide has a high binding affinity for integrin *α*_v_ *β*_3_, which is highly expressed in U87 MG tumors, but has low expression in PC‐3 tumors. In U87MG/PC‐3 tumor‐bearing mice, the radiolabelled peptide showed selective tumor accumulation and clear SPECT/CT (single photon emission computed tomography combined with computed tomography) imaging at 24 h, indicating TBD‐DO3A's utility for directed radiotheranostics.

**SCHEME 8:**
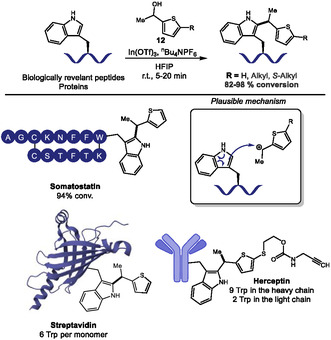
Alkylation of Trp residues with thiophene‐ethanol derivatives.

The formation of C—C bonds is also of great interest for bioconjugation given the stability of those bonds under biological conditions. In 2024, Ohata and coworkers used HFIP as a nonaqueous medium for a fast and highly chemoselective Trp labeling using In(OTf)_3_ as a Lewis acid catalyst and thiophene‐ethanol derivatives **12** as alkyl donors (Scheme 8) [48]. They achieved the dehydrative *C*2‐alkylation of tryptophan in 5 biologically relevant peptides, like somatostatin and Luteinizing hormone‐releasing hormone (LHRH), with 82%–98% conversion and typically within 5–20 min. They applied the transformation to proteins like streptavidin, lysozyme, myoglobin, and the antibody Herceptin, with quantitative modification achieved within minutes. Authors also demonstrated the utility of the method for the introduction of a ^18^F‐labeled thiophene‐derivative for radiofluorination purposes. Reaction proceeded through Lewis acid activation of thiophene‐ethanol, with benzylic carbocation formation stabilized by HFIP, followed by electrophilic aromatic substitution. The reaction exhibited exceptional selectivity for Trp over all other canonical amino acids, as confirmed by absence of reaction in Trp‐lacking peptides and proteins, as well as MS/MS analysis of modified substrates. Interestingly, the authors also showed some level of oxidation that is not present in Trp‐lacking proteins, indicating this oxidation occurs only at Trp sites. Selectivity was attributed to HFIP's unique solvation properties, that stabilized cationic intermediates while potentially suppressing Tyr and His reactivities *via* H‐bonding. A key limitation was the dependence on HFIP solventtodium, although the authors showed that short‐term treatment of some proteins like lysozyme was not detrimental for its structure.

Samples of the monoclonal antibody Herceptin labeled with an alkyne‐modified thiophene‐ethanol reagent were submitted to cycloaddition to an azide‐containing fluorophore installed in a blot membrane, allowing the observation of fluorescent signals indicating successful tagging of both the heavy and the light chains of the antibody.

A few months later, Ohata's group developed an indium‐free variant of this work, using sulfonic acid‐based imidazolium salts as acidic ionic liquid catalysts, enabling efficient modification in Trp containing somatostatin peptide and HEK293T cell lysates [49]. The authors retained HFIP as the solvent and the core thiophene‐ethanol reagent design, since better leaving groups led to loss of Trp selectivity. This advance expands the methodology's utility for studying redox or metal‐sensitive biological samples.

**SCHEME 9:**
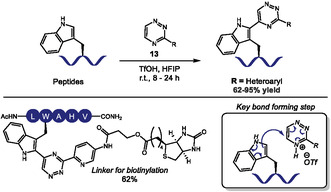
TfOH‐promoted C2‐heteroarylation of Trp residues in peptides.

Xiong and coworkers reported a metal‐free tryptophan‐selective *C*2‐heteroarylation in peptides with triazine derivatives **13** in HFIP TfOH activation (Scheme 9) [50]. The reaction was applied to small molecules and complex peptides, like growth hormone‐releasing peptide‐2 (GHRP‐2) and leuprorelin, achieving 62%–95% LC‐MS yields with good chemoselectivity for Trp even in the presence of nucleophilic residues like Cys, Lys, His, Tyr, and N‐terminal amines. Triflic acid activation of the triazines enables electrophilic aromatic substitution at the indole *C*2‐position, while HFIP stabilizes cationic intermediates and acts as proton shuttle to accelerate aromatization. The acidic conditions suppress competing reactions from other residues, though reaction with Nterminal Trp residue, which is usually hindered in these conditions, was not explicitly tested. The installed triazine can serve as handle for further bioorthogonal inverse electron‐demand Diels‐Alder (IEDDA) reactions with trans‐cyclooctene derivatives, as demonstrated with one peptide product.

**SCHEME 10:**
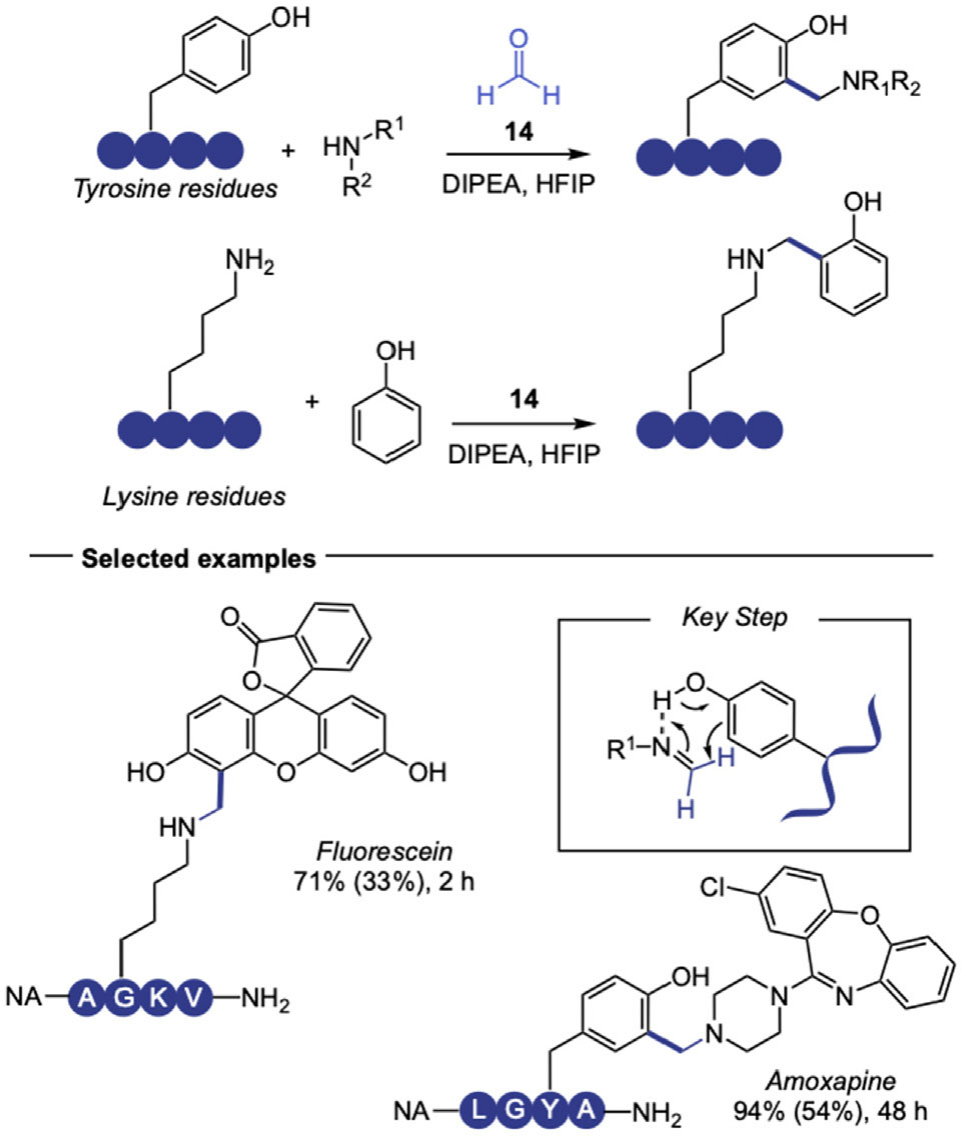
Condensation of tyrosine and lysine to amine and phenol containing bioactive molecules respectively, via Betti reaction.

Following the pioneering work of Francis’ group on the three‐component Mannich‐type bioconjugation of tyrosine [51], in 2024 Chen and coworkers explored it as a strategy to efficiently crosslink diverse biomolecules via their endogenous phenol and amine moieties (Scheme 10) [52]. The transformation, carried out in the presence of formaldehyde (**14**) and HFIP as solvent, showed strong dependence on HFIP. This effect was attributed to the solvent's ability to form aggregates that approximate the reaction partners through multiple intermolecular interactions. This platform was used for the late‐stage functionalization of Tyr and Lys residues, crosslinking these amino acids with various peptides, bioactive molecules, and fluorophores in good to excellent yields. Despite its broad functional group tolerance, the method faces selectivity challenges in the presence of arginine and cysteine, whose guanidine and thiol side chains compete with phenols in the nucleophilic addition step.

**SCHEME 11:**
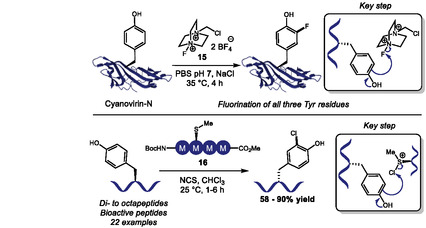
Halogenation of Tyr residues through electrophilic aromatic substitution.

Recently, some Tyr halogenation strategies for bioconjugation were also reported. In 2024, our group reported the first late‐stage electrophilic fluorination of tyrosine residues in proteins (Scheme 11, top) [53], building on the reactivity of Selectfluor (**15**) to fluorinate electron‐rich aromatic rings [54, 55]. Using aqueous phosphate buffer media, the authors enabled C–F bond formation through an electrophilic aromatic substitution mechanism. Within the amino acid scale, the authors demonstrated that Tyr can be selectively fluorinated at the *ortho* position even in the presence of other aromatic amino acids like His, Trp, and Phe. Structural similarity hindered purification, and despite a good conversion, the pure product was obtained only in 29% yield, which underscores the importance of late‐stage modification in native proteins as a more practical approach. The methodology was validated on Cyanovirin‐N (CVN), fluorinating all three tyrosine residues (Y11, Y31, Y102) of the protein chemoselectively within 4 h of reaction at 35°C. Tryptophan, cysteine, and methionine residues underwent oxidation, which was partially addressed by adding methionine as a sacrificial agent. The authors also described some level of difluorination in some residues. Fluorinated CVN retained mannose‐binding affinity in comparison with unreacted samples, confirming structural integrity and endorsing the method as an interesting strategy to attach ^19^F‐NMR probes for protein dynamics studies.

Building on the growing interest in peptide‐based catalysts [56] and in previous aromatic halogenations using catalytic sulfide and *N*‐halosuccinimide sources [57, 58, 59], in 2025 Maji and coworkers published the design of a methionine‐based oligopeptide catalyst (**16**) to facilitate the late‐stage electrophilic chlorination of tyrosine residues in peptides and electron‐rich arenes using *N*‐chlorosuccinimide (NCS) under mild conditions (Scheme 11, bottom) [60]. While the scope is broad, encompassing 21 examples of tyrosine‐containing di‐ to octapeptides, achieving halogenated products in 58%–90% yield, it lacked selectivity studies against other nucleophilic aromatic amino acids, like Trp or His. The method had very low catalyst loadings, down to 0.25 mol%, and gram‐scale applicability. Some remarkable limitations included inefficacy in predominantly aqueous systems and the need for primary amine protection. Mechanistic studies confirmed methionine sulfide acted as a nucleophilic catalyst, abstracting the halogen atom from NCS, generating a halonium ion. Kinetic profiling also showed reaction rate increases proportionally with the number of methionine sites in the catalyst.

## 3 Addition Reactions

Addition reactions have been explored as a key step in protein labeling, giving access to the efficient conjugation of a wide variety of biomolecules through versatile linkers. In this context, carbonyl chemistry, boron chemistry, and cycloadditions have emerged as the most widely applied synthetic approaches [61], making significant contributions to biochemistry and medicinal research.

Biomolecule labeling has found wide applicability in cell imaging, contributing significantly to the understanding of structural biology [62]. The conjugation of biostructures with heavy‐metal nanoparticles has offered major advantages for cellular studies compared to traditional dyes, due to their inherent optical properties [62, 63]. Gold nanoparticles, in particular, have been extensively employed in this field because of their chemical inertness, nontoxic nature, high electron density, and strong optical absorption [63, 64]. Common conjugation strategies take advantages of gold nanoparticle interactions with thiol groups and also include N‐hydroxysuccinimide chemistry and click chemistry [63, 64, 65].

**SCHEME 12:**
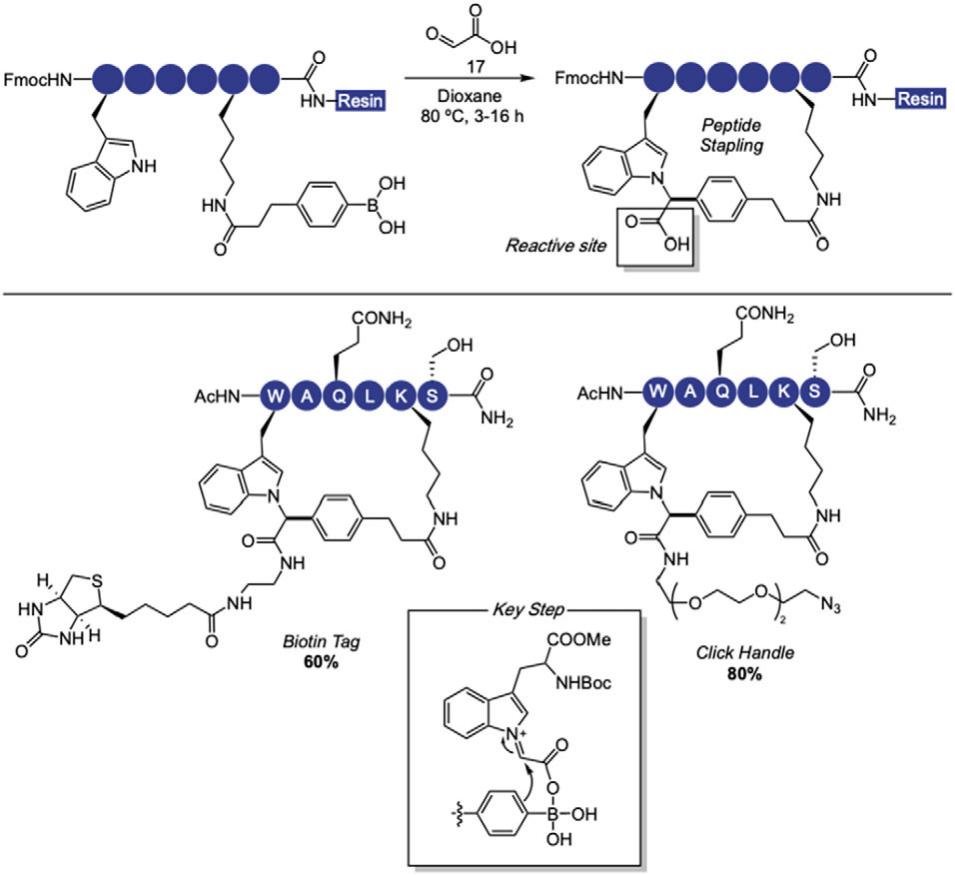
Peptides late‐stage functionalization and cyclization enabled by glyoxylic acid via multicomponent Petasis reaction.

In 2023, Krajcovicova and Spring reported a tryptophan‐based multicomponent Petasis reaction that enables simultaneous peptide cyclization and late‐stage functionalization using glyoxylic acid (**17**) as the electrophile [66]. Although the Petasis reaction had previously been applied to peptide diversification targeting N‐terminal proline, *N*‐methyl lysine [67], and ornithine [68], it had not yet been explored for tryptophan labeling. The methodology exhibits high chemoselectivity among various nucleophiles, although primary amines required protection to prevent undesired side reactions (Scheme 12). The authors successfully applied this strategy to cyclize a broad range of peptides, including biologically relevant sequences, while introducing strategically chosen functional tags such as fluorescent groups. The approach was compatible with commonly protected peptides, yielding more stable macrocyclic versions and demonstrating its potential for peptide‐based drug development and probe design.

**SCHEME 13:**
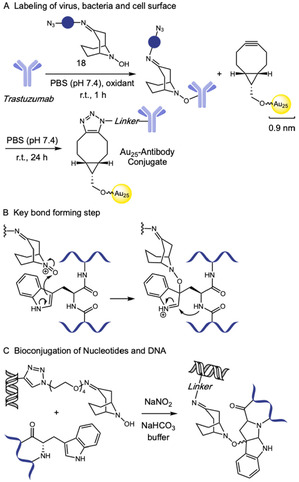
Tryptophan selective bioconjugation using hydroxylamine enables Au_25_‐antibody conjugates formation.

In the same year, Kanai and coworkers introduced an Au_25_–antibody conjugate as a promising electron‐dense probe to enhance resolution in cryogenic electron microscopy (cryo‐EM) and cryo‐electron tomography (cryo‐ET) of biological structures [69]. The researchers adjusted their already developed pH‐neutral bioconjugation protocol targeting tryptophan residues [70], enabling the installation of the *N*‐hydroxy‐9‐azabicyclo [3.3.1]nonane derivative (N_3_‐ABNOH, **18**), under aqueous, additive‐free, and pH‐neutral conditions, particularly suitable for acid‐sensitive proteins such as antibodies. The azide‐modified biomolecules were then coupled to Au_25_ nanoclusters through a copper‐free click reaction. This method was applied to the monoclonal antibody trastuzumab, whose conjugates were successfully visualized by cryo‐EM using less than half the usual sample amount (Scheme 13, part A). This strategy offers a powerful, nongenetic approach to enhance structural resolution in cryo‐EM and cryo‐ET, holding significant promise for biomedical applications.

In 2024, Spampinato and coworkers reported the novel application of ABNOH in the synthesis of modified nucleotides [71]. In this study, uridine nucleotides were functionalized via CuAAC reaction with ABNOH‐PEG_4_‐N_3_ and successfully incorporated into DNA by polymerases. The resulting modified DNA probes could react selectively with Trp residues under mild oxidative conditions (Scheme 13, part C). The methodology was validated through the conjugation of these DNA probes with peptides and with the DNA‐binding regulatory protein *bs*GntR, which contains a Trp residue at the DNA interface, forming stable tricyclic adducts. The reactions were performed in different media (water, physiological, and basic buffers), with best yields at pH 10. Conversions of up to 85% were obtained in the presence of NaNO_2_, but also satisfactory yields (20%–50%) were reached in its absence, avoiding potential side effects of DNA deamination.

Activity‐based protein profiling (ABPP) is a proteomics strategy widely applied to investigate the functional sites of proteins under physiological conditions [72, 73]. While numerous covalent probes have been developed to target cysteine residues [74, 75, 76], histidine side chains have remained largely unexplored due to their comparatively lower nucleophilicity.

Inspired by the facile autoxidation of methionine residues to methionine sulfoxides observed during mass spectrometry analyses, Chang and Toste developed a methodology termed redox‐activating chemical tagging (ReACT) [77, 78]. This approach exploits a strain‐driven sulfur imidation using N‐carbamoyl or N‐carboxyl oxaziridines to selectively conjugate methionine residues.

**SCHEME 14:**
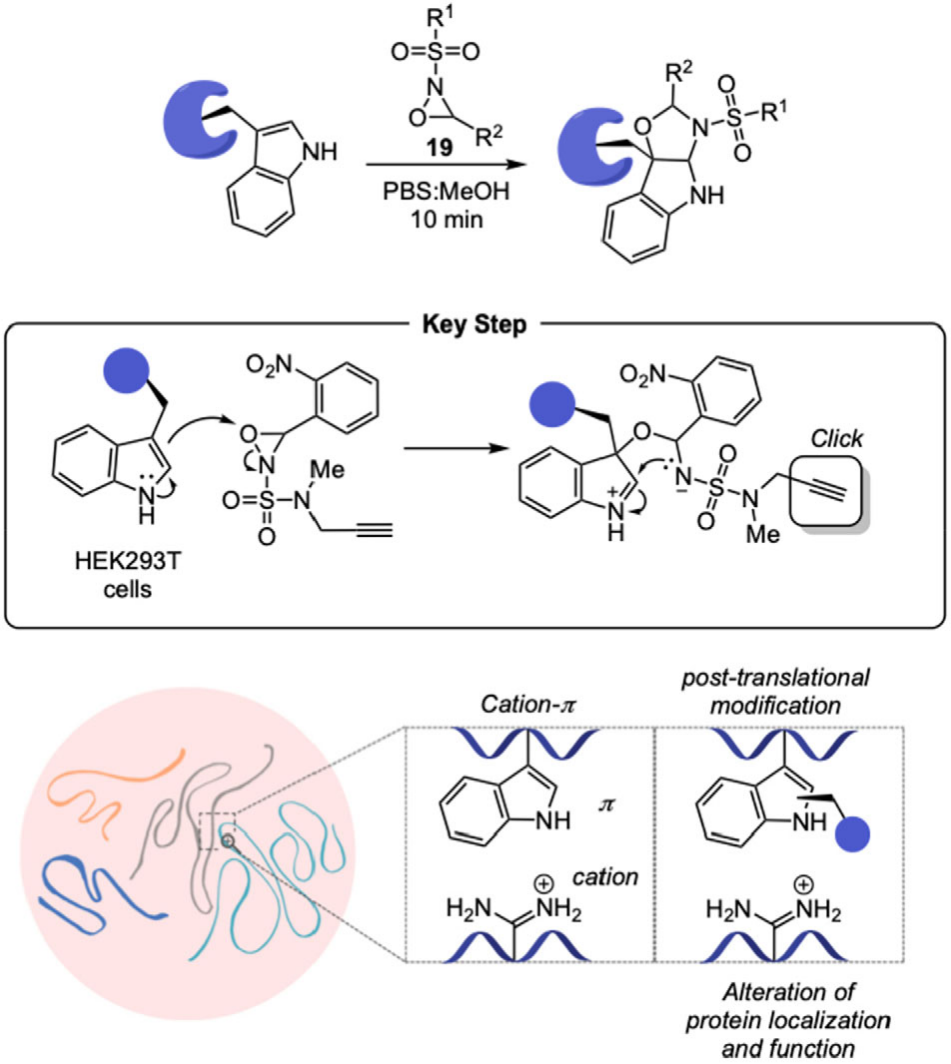
Trp‐Click approach to selective conjugate tryptophan residues to biologically relevant structures.

Following this pioneering work, the authors further investigated the electronic and chemical properties of the reagent to enable the conjugation of additional amino acid side chains. In 2024, they developed a remarkable strategy for the selective bioconjugation of tryptophan inspired by the oxidative cyclization found in the biosynthesis of indole alkaloids [79]. Although tryptophan showed neglegible reactivity toward, *N*‐alkoxycarbonyl oxaziridines in latter reports on methionine functionalization, by exploring the higher electrophilicity of *N*‐sulfonyl oxaziridines **19**, the authors established a methodology named Trp‐CLiC, which enables selective oxidative cyclization at tryptophan residues even in the presence of other nucleophilic amino acids (Scheme 14). This approach proved highly efficient for the site‐selective installation of biologically relevant functional groups such as pharmacophores and fluorescent probes, opening new possibilities for molecular tracking, functional modulation, and targeted delivery. To demonstrate the broad applicability of this method, the authors developed a proteome platform capable of mapping hyper‐reactive tryptophan residues. The distribution profile of these reactive sites revealed a strong association with phase‐separated cellular compartments. Further analysis showed that tryptophan‐mediated cation–*π* interactions play a key role in the formation and stabilization of these membraneless organelles and that disease‐associated mutations or post‐translational modifications that compromise these interactions can alter protein localization and misregulate subcellular organization.

**SCHEME 15:**
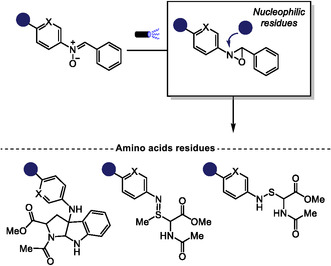
Light‐driven late‐stage functionalization of nucleophilic amino acid residues mediated by in situ oxaziridine formation.

In 2025, Toste and coworkers explored the light‐induced isomerization capability of *N*,*α*‐diaryl nitrones as a strategy to access reactive oxaziridine intermediates for late‐stage functionalization of amino acids, peptides, and proteins [80]. They demonstrated that this class of nitrones undergoes efficient photoisomerization, with the process being strongly influenced by the electronic properties of substituents on the *α*‐aryl ring. Notably, increased electron density on this aromatic ring not only enhanced photoisomerization but also promoted rapid degradation of the oxaziridines in solution. By electronically tuning these substituents, the authors succeeded in generating oxaziridines derivatives with sufficiently long lifetimes to engage in bioconjugation reactions. These intermediates enabled the efficient functionalization of cysteine, methionine, and tryptophan residues, and to a lesser extent, serine. The resulting bioconjugation products were stable in aqueous media. Encouraged by these results, the authors demonstrated the potential of this light‐driven strategy for mapping reactive amino acid residues within the mammalian proteome, highlighting its utility in studying post‐translational modifications and biomolecular target identification (Scheme 15).

## 4 Heteroatom as Nucleophile

Recently, transformations targeting the nucleophilic heteroatoms of Trp, Tyr, and His have been reported, engaging them in reactions such as substitution at sulfur and conjugate additions.

**SCHEME 16:**
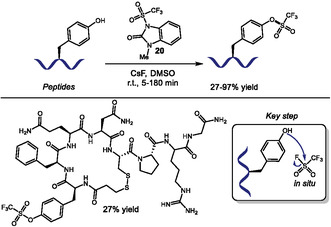
Tyr‐selective O‐triflation of peptides.

In 2023, Schiesser and coworkers reported a mild method for converting phenols to aryl O‐triflates using a triflate‐imidazolone **20** as donor with CsF in DMSO at room temperature (Scheme 16). Their scope included small molecules and peptides, with yields ranging from 27%–97% [81]. They achieved Tyr‐selective O‐triflation in peptides containing nucleophilic residues like threonine (Thr), His, arginine (Arg), Met, and Trp, without side‐reactions at disulfide bonds. However, the authors showed that cysteine residues are incompatible with this reaction, leading to undesired adducts, and free amines or carboxylic acids require acid or base additives for optimal yields, respectively. The proposed mechanism involves in situ generation of trifluoromethanesulfonyl fluoride as the active species, which enhances functional group tolerance by avoiding direct reaction with sensitive side chains unlike previous methods using *N*‐phenyltriflimide (PhNTf_2_) [82].

**SCHEME 17:**
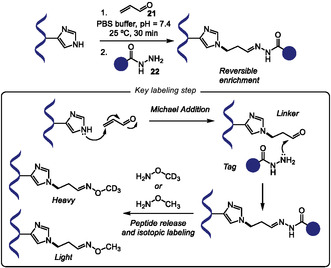
Acrolein‐based histidine labeling.

In the same year, Li and coworkers introduced a chemical proteomics platform designed to quantitatively and site‐specifically analyze the reactivity of histidine residues across the human proteome. The researchers explored histidine nucleophilicity to tag these amino acids residues with acrolein (**21**) probes through Michael addition [83]. The modified amino acid is later enriched with hydrazine moieties **22**. The reversibility of this chemistry allows isotopic labeling during peptide release step avoiding further isotopic linker preparation for quantitative profiling. An advantage of this method includes the addition of a small tag compared with traditional click chemistry tags, which could enhance labeling range due to lack of steric hindrance. Since cysteine and lysine showed to be chemical competitors for ACR‐based labeling, site‐specificity was achieved by combining *N*‐ethylmaleimide alkylation step prior to reaction with ACR probes (Scheme 17). This platform enabled the quantification of over 8,200 histidine residues in the human proteome, pointing to the discovery of 1107 new proteins not included in the Drugbank database. This technology opens path to discovering new druggable targets for precise therapy.

**SCHEME 18:**
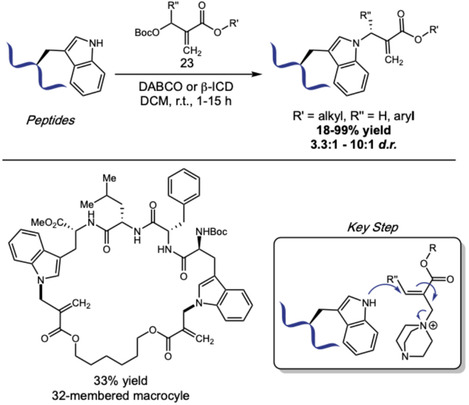
Allylation of Trp residues within peptides, and application in peptide–peptide coupling and macrocyclization.

Inspired by Chen's work on *N‐*allylic alkylation of Indoles with Morita–Baylis–Hillman carbonates (MBHCs) [84], in 2024, Sun, Wang, Xu, and coworkers reported the organocatalytic *N*1‐allylation of Trp using tertiary amine catalysis (Scheme 18) [85]. The transformation employs DABCO‐activated MBHCs as allylating reagents. The scope accommodates diverse Trp‐containing protected di‐ to heptapeptides, including bioactive sequences like endomorphin‐1, and diverse MBH carbonates **23** bearing esters, lipids, polyethylene glycol (PEG), alkynes, fluorophores, glycosyl groups, and natural products, with yields ranging from 58% to 99%. Reaction proceeds through tertiary amine activation of MBHC, followed by nucleophilic attack of Trp nitrogen. Selectivity studies confirmed exclusive *N*1‐regioselectivity over other indole positions and preferential modification of Trp over Tyr and His. Loss of chemoselectivity was observed when Lys, Arg, Ser, and Cys residues were present, unless properly protected. The authors also observed that using bifunctional *β*‐Isocupreidine as chiral tertiary amine catalyst they could achieve an assymetric version of this reaction, with varying diastereoselectivities. The method also enabled a remarkable peptide macrocyclization with construction of 17–35‐membered rings via intramolecular allylation or peptide stapling between two Trp units and a MBH diester. As a proof of concept, the authors engaged one of these modified substrates in a peptide–peptide conjugation through thia‐Michael addition of a cysteine‐containing partner, showing the utility of this methodology for peptide modification and peptide‐drug conjugate design.

**SCHEME 19:**
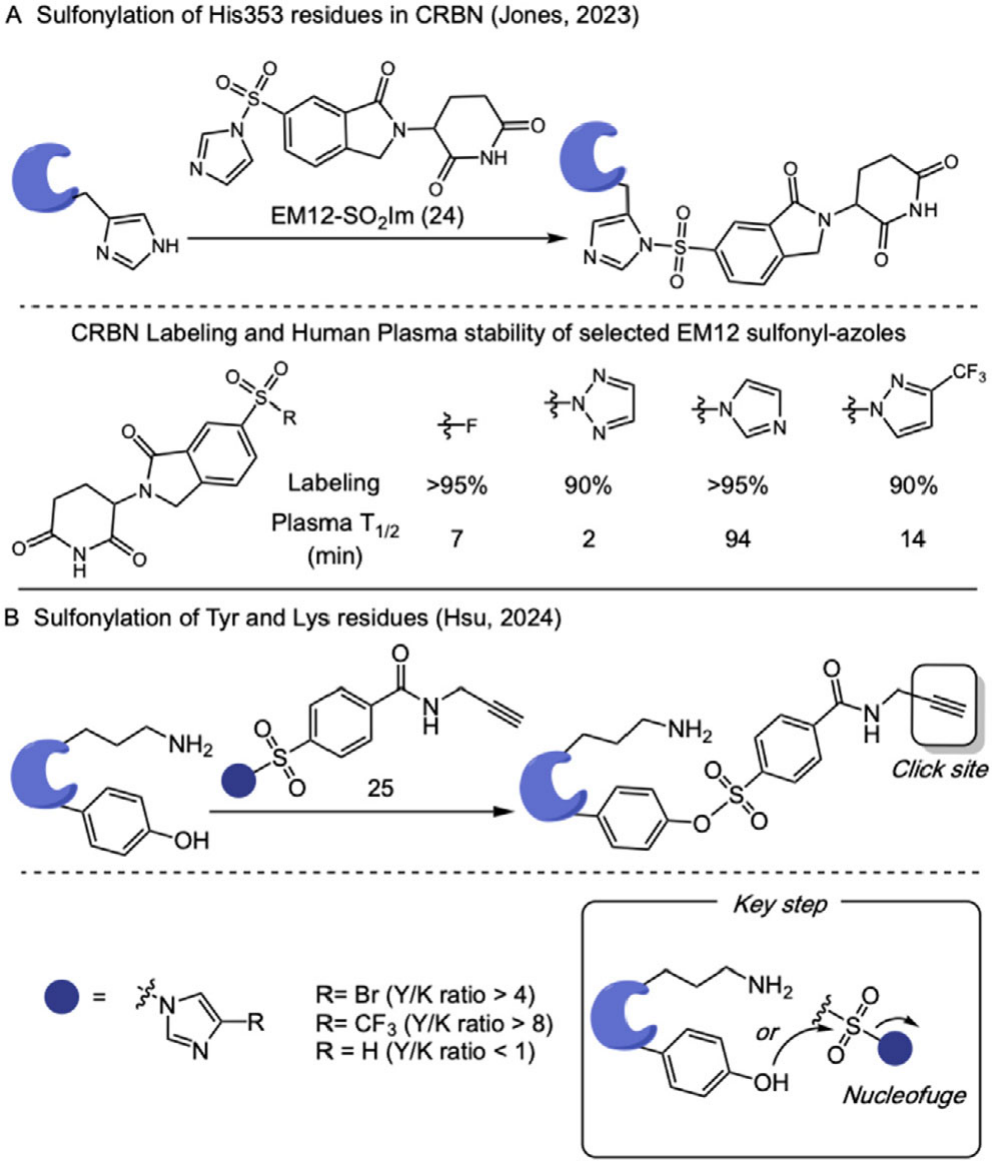
Sulfonyl‐azole‐mediated labeling of proteins.

Sulfonyl‐triazole exchange (SuTEx) chemistry allows the use of tunable triazole leaving groups to promote the sulfonylation of biomolecules [86]. However, they usually present low stability in plasma. In their work on covalent stapling of Cereblon (CRBN), Jones and coworkers explored the use of some sulfonyl‐azole derivatives of EM12, a molecular glue degrader, to covalently bind His353 residues in the sensor loop [87]. They systematically tuned the electrophilicity of the warheads, transitioning from sulfonyl fluorides and triazoles to less electrophilic sulfonyl‐diazoles (SuDEx), particularly sulfonyl imidazoles. The optimized compound EM12‐SO_2_Im (**24**) achieved effective stapling of CRBN in a closed conformation and displayed increased plasma stability compared to other evaluated sulfonyl derivatives (Scheme 19A).

In 2024, Hsu and coworkers further explored this platform for selective mapping tyrosine and lysine residues across the proteome [88]. The authors leveraged the leaving group ability of azole derivatives to design tunable sulfonyl‐azole based electrophile **25** for site‐selective protein labeling. Among a range of synthesized five‐membered sulfur–azole compounds, sulfonyl‐imidazoles exhibited an optimal balance of reactivity and stability under biological conditions. Furthermore, by modulating the electronic properties of the imidazole scaffold, the researchers were able to modulate both the overall labeling efficiency and the selectivity between tyrosine and lysine residues (Scheme 19B). This strategy provides a valuable tool for further development chemoproteomic profiling, drug discovery, and functional annotation of proteins in complex biological systems.

**SCHEME 20:**
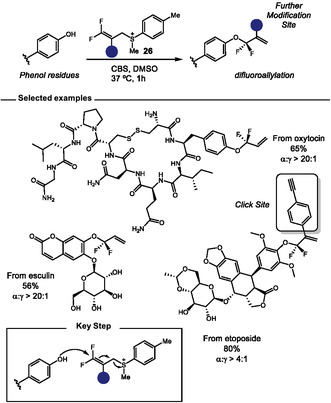
O‐Difluoroalkylation of phenol containing bioactive compounds including tyrosine residues in peptides.

In 2024, Zhang and coworkers presented an efficient method for the *O‐*difluoroalkylation of phenol‐containing bioactive molecules, such as tyrosine residues (Scheme 20) [89]. They used a bench‐stable, but highly active, 3,3‐difluoroallyl sulfonium salt **26,** previously developed by the group [90]. The reaction proceeds under biocompatible mildly basic aqueous conditions. The authors showed that enolizable carbonyl groups and chiral centers, typically sensitive to racemization under basic conditions, were compatible with the carbonate‐buffered saline (CBS buffer) used in the optimized protocol. The method also exhibited high chemoselectivity toward phenol groups, enabling the selective modification of tyrosine in the presence of other nucleophilic amino acid residues, unprotected carbohydrates, and nucleosides. Furthermore, the incorporation of vinyl groups into biomolecules allows for subsequent valuable modifications, paving the way for future applications in medicinal chemistry and chemical biology.

## 5 Biocatalysis

Biocatalysis has emerged as a valuable tool for biomolecule conjugation, offering key advantages such as exceptional selectivity and compatibility with physiological conditions. Despite challenges related to limited substrate scope, versatility, and the frequent requirement for recognition tags in most applications, the field has seen significant progress. Advances in protein engineering have expanded the range of accessible transformations, broadening the applicability of biocatalytic methods in chemical biology [91, 92].

Indole prenyltransferases (IPTs) represent a broad class of enzymes that catalyze the prenylation of indole‐containing substrates at distinct positions of the aromatic scaffold [93, 94]. While synthetic methods have achieved such functionalizations [95, 96, 97], they often face limitations in chemoselectivity and generate undesired side products, making biocatalytic approaches more attractive alternatives.

**SCHEME 21:**
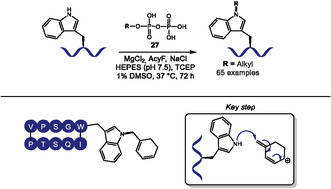
Tryptophan functionalization via biocatalyzed carbocations generation.

Cyanobactin prenyltransferases have been used to incorporate prenyl or geranyl groups in several peptides. However, these enzymes are usually extremely sensitive, only allowing the transfer of natural isoprene units. In 2023, Houssen and coworkers addressed this issue, showing that the N1‐tryptophan prenyltransferase AcyF accepts synthetic alkyl pyrophosphate analogs, promoting the late‐stage functionalization of peptides [98]. Their methodology allowed the enzymatic transfer of 6 unnatural pyrophosphate donors **27**, including diene and azide‐containing structures, onto the indole nitrogen of Trp in a cyclic model peptide. The mechanism involves biocatalyzed generation of stabilized carbocations due to loss of pyrophosphate, following nucleophilic attack of indole nitrogen. The authors showed strict amino acid specificity for N1‐Trp, but donor alterations due to carbocation fragmentation or rearrangement to resonance‐stabilized intermediates led to complex mixtures or unexpected products. After enzymatically installing a diene, the authors proposed a bioorthogonal fluorescent tagging through an inverse electron‐demand Diels–Alder (IEDDA) reaction with a tetrazine, showcasing the utility of the methodology for peptide tracking (Scheme 21).

**SCHEME 22:**
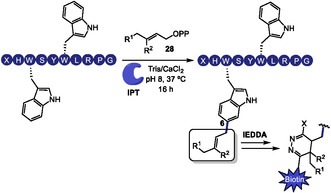
IPT‐mediated prenylation of tryptophan residues at C6 position.

In 2024, Elshahawi and coworkers reported a selective late‐stage chemoenzymatic method for the functionalization of tryptophan residues in cyclic and linear biorelevant peptides [99]. By employing an indole prenyltransferase (IPT) and a range of alkyl diphosphates **28**, the authors achieved site‐selective alkylation at the C6 position of the indole ring in tryptophan. Notably, the transformation occurs selectively at a single tryptophan residue, even when multiple tryptophans are present within the peptide sequence and preferentially targets tryptophans located near the peptide termini. The installed alkyl handle enables efficient bioorthogonal tetrazine ligation via an inverse electron‐demand Diels–Alder (IEDDA) reaction with a biotin‐conjugated tetrazine probe (Scheme 22). This two‐step strategy allows precise and biocompatible tagging of complex peptides, providing a versatile platform for C–H functionalization with chemical biology applications.

**SCHEME 23:**
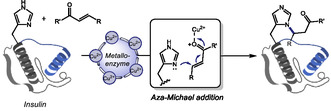
Site‐specific histidine functionalization through Michael addition enabled by a human ferritin‐based metalloenzyme.

In 2024, Wang and coworkers reported the engineering of a human ferritin‐based metalloenzyme capable of achieving site‐specific histidine modification in peptides and proteins via aza‐Michael addition [100]. By incorporating noncanonical histidine analogs into key positions of the ferritin heavy chain and loading the protein cage with Cu(II), the authors created an artificial metalloenzyme scaffold that catalyzes *N*‐substitution of single histidine residues (Scheme 23). The system was successfully applied to a panel of eight peptide and protein substrates ranging from 10 to 607 amino acids in length. For insulin, a protein containing two histidine residues, the authors achieved exclusive modification of histidine at position 5 of the B‐chain. This high level of selectivity was enabled by fusing an insulin‐targeting peptide directly to the engineered ferritin scaffold, guiding the modified‐enzyme to engage specifically with the desired histidine site. Upon addition of this targeting element, it was observed an improvement in conversion yields from 46% to 100%, despite the decrease in the turnover number of the engineered metalloenzyme. That way, this work establishes a powerful chemoenzymatic platform to modify proteins at specific histidine residues, adding invaluable contributions to protein bioconjugation toolbox for potential in vivo applications.

Among flavin‐dependent halogenases, tryptophan halogenases form a well‐characterized subclass that catalyzes the regioselective halogenation of the indole ring in tryptophan under mild conditions [101]. These enzymes have been extensively studied and applied to generate halogenated tryptophan derivatives with diverse regioselectivities, thereby expanding the accessible chemical space [102, 103, 104, 105]. However, when it comes to the halogenation of biomacromolecules such as peptides, current strategies still rely predominantly on the prior synthesis of halogenated amino acid building blocks, which are then incorporated into peptides during solid‐phase or ribosomal synthesis, rather than direct enzymatic modification of the macromolecular scaffold.

**SCHEME 24:**
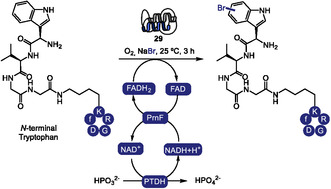
ThaI (**29**)‐mediated N‐terminal tryptophan bromination.

In 2023, Sewald and coworkers investigated the substrate flexibility of the tryptophan 6‐halogenase ThaI (**29**) to establish an enzymatic platform for the site‐selective bromination of tryptophan‐containing peptides (Scheme 24) [106]. The method enables efficient C6‐selective bromination of both L‐ and D‐tryptophan residues located at the N‐terminus of peptides, with substrates of up to five residues in length. Incorporation of phosphite dehydrogenase for in situ NADH regeneration significantly enhanced total turnover numbers, improving overall reaction efficiency. To demonstrate its applicability in bioconjugation, a recognition motif bearing an N‐terminal tryptophan was conjugated to an RGD peptide and successfully brominated under the developed conditions.

**SCHEME 25:**
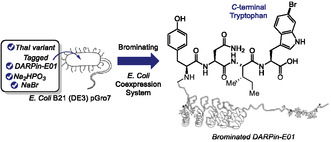
Bromination of C‐terminal tryptophan residues enabled by a ThaI variant using a developed *E. coli* coexpression system.

In 2024, Sewald and coworkers engineered a variant of Thal capable of selectively brominating tryptophan residues located at the C‐terminus of short peptide tags fused to target proteins [107]. Through a peptide library screening, they identified an optimal tag sequence (Y–N–I–W) that enabled efficient and site‐selective enzymatic bromination in vivo using a coexpression system in *E. coli.* The engineered enzyme showed a clear preference for bromination over chlorination at the C6 position of the indole ring (Scheme 25). To demonstrate the utility of the brominated proteins, the authors applied them in palladium‐catalyzed Suzuki–Miyaura cross‐coupling with boronic acids under mild aqueous conditions. This work demonstrates a useful strategy for the site‐specific modification of proteins at their C‐terminus via enzymatic bromination.

Tyrosinases are widely studied and versatile conjugation tools [108, 109, 110, 111, 112]. These oxidoreductases catalyze the oxidation of phenols to O‐quinones, highly electrophilic intermediates that can undergo a variety of chemical transformations, including Mannich reactions, Michael additions, and cycloadditions [110, 113]. Therefore, these enzymes exhibit significant potential as a platform for the development of new chemical biology technologies.

**SCHEME 26:**
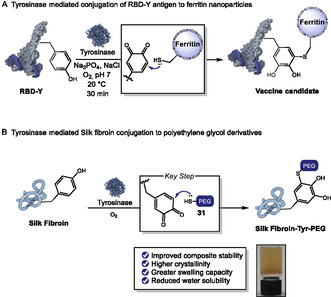
Tyrosinase‐mediated conjugation of RBD‐Y antigen to ferritin nanoparticles.

In 2024, Roldão and coworkers introduced a tyrosinase‐mediated bioconjugation strategy as a promising tool for vaccine development. In this approach, *Agaricus bisporus* tyrosinase was employed to conjugate the receptor‐binding domain of the SARS‐CoV‐2 spike protein, modified with a C‐terminal tyrosine (RBD‐Y), to native cysteine residues exposed on the surface of ferritin nanoparticles [114]. The transformation involves the selective oxidation of the tyrosine residue to an *o*‐quinone electrophile in the presence of molecular oxygen, followed by a site‐selective 1,6‐Michael addition with surface‐exposed thiols on ferritin (Scheme 26A). This enzymatic strategy offers a simple and efficient alternative to traditional genetic fusion methods for multivalent antigen display, with potential for broader applications in biotechnology.

Liu and coworkers also explored this strategy of site‐selective tyrosine conjugation, mediated by tyrosinase, to functionalize silk fibroin proteins with various polyethylene glycol (PEG) derivatives **31** (Scheme 26B) [115]. This biomimetic approach enabled the conjugation of dopaquinone, formed by tyrosinase oxidation of tyrosine residues, to a thiol‐functionalized PEG via Michael addition, effectively avoiding random crosslinking. The resulting PEG‐SH‐conjugated fibroin formed hydrogels with enhanced physicochemical properties. Notably, the conjugation increased the *β*‐sheet content, giving rise to extended, resistant *β*‐sheet‐like structures with uniform and small pores. These structural and molecular rearrangements resulted in improved composite stability, higher crystallinity, greater swelling capacity, and reduced water solubility, highlighting the potential of this transformation for advanced biomaterials applications.

**SCHEME 27:**
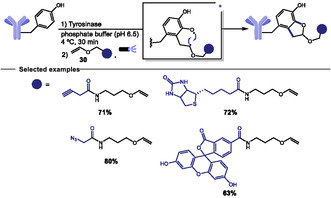
Photoenzymatic strategy to specific conjugate tryrosine residues to biologically relevant molecules through vinyl ethers addition.

In 2023, Xia and coworkers developed a photoenzymatic strategy that combines *A. bisporus* tyrosinase with a blue‐light–induced photoaddition of vinyl ethers **30** to enable site‐selective conjugation of C‐terminal tyrosine‐tagged biomolecules under mild aqueous conditions (Scheme 27) [116]. This methodology was extended to the selective modification of a unique tyrosine residue (Y296) in the Fc domain of human IgG antibodies, allowing the installation of environmentally stable linkers for the formation of antibody–drug conjugates (ADCs) with a potent antimitotic agent. The strategy was also applied to a HER2‐specific nanobody engineered with a C‐terminal tyrosine tag, enabling its fluorescent labeling and subsequent conjugation to THP1 cells, a human monocytic leukemia cell line with macrophage‐like properties. The resulting nanobody–cell conjugates exhibited enhanced recognition and killing of HER2‐positive SKOV3 cancer cells via antibody‐dependent cellular cytotoxicity (ADCC).

**SCHEME 28:**
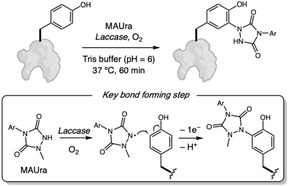
Laccase‐catalyzed oxidation of MAUra for tyrosine labeling.

Laccases also belong to the oxidoreductase class which catalyzes oxidation reactions by reducing molecular oxygen and, as well as tyrosinases, serve as a tool in protein bioconjugation [117, 118]. Leveraging this reactivity, Sato and coworkers [119] reported the use of laccase to selective oxidize a labeling reagent 1‐methyl‐4‐arylurazole (MAUra) without oxidizing the Tyr residues (Scheme 28). Kinetic and radical scavenger experiments suggest a mechanistic pathway involving the formation of MAUra radicals followed by its addition to Tyr phenolic ring and further one‐electron oxidation. Although the reaction conditions also modified Trp residues, Tyr was labeled preferentially and this problem is minimized in proteins, where tryptophan's exposure is minimal. This methodology was successfully applied to tyrosine‐containing bioactive peptides, such as oxytocin, thymopentin, kisspeptin‐10, and cyc(RGDyK), as well as complex proteins including BSA, glucose oxidase, and trastuzumab, under mild conditions, avoiding oxidative side reactions. Proteomic analysis identified over 3,000 unique modification sites, predominantly on surface‐exposed Tyr. Compared to classical reagents such as PTAD, the laccase and MAUra system offers higher stability, avoids oxidative by‐producs, and enables modification of internal Tyr residues, providing a green and practical approach for the functional labeling of native peptides and proteins.

## 6 Transition Metal Catalysis

Transition metal catalysis has emerged as a powerful strategy for enabling diverse synthetic transformations in complex systems, including the modification of biomolecules. In the last 3 years, palladium, rhodium, iridium, and ruthenium have been predominantly employed in C—C bond formation reactions, whereas copper distinguishes itself as the preferred catalyst for the functionalization of heteroatom‐containing amino acid side chains. Palladium is the most widely used catalyst for both innate and directed C–H activation reactions of aromatic amino acids residues, while Rh, Ir, and Ru are typically used only in the presence of directing groups. Despite these advances, the application of transition metal catalyzed transformations to more complex systems such as proteins remains underexplored.

### 6.1 Palladium

Transition metal‐catalyzed C–H functionalization at the C2 position of the indole ring has been extensively investigated, as this site is inherently the most reactive toward metalation [120]. The first report of undirected Pd‐catalyzed C2 arylation of tryptophan in small peptides was reported by Albericio and Lavilla in 2010 [121], using aryl iodides as coupling partners. Later, other more electrophilic coupling partners were also applied allowing C2 arylation under neutral and aqueous conditions and with minimal waste [122].

**SCHEME 29:**
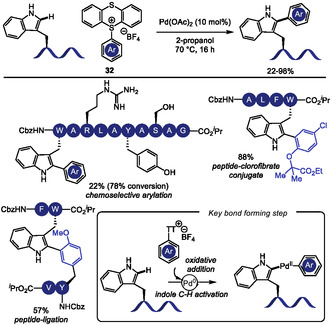
Pd‐catalyzed C–H arylation of tryptophan C2 position using arylthianthrenium salts.

In 2023, Ackermann and coworkers [123] leveraged this innate reactivity of indole C2 position to achieve a Pd(II)‐catalyzed undirected C–H activation of Trp‐containing oligopeptides using arylthianthrenium salts **32** [124] as readily accessible arylating agents (Scheme 29). The reaction proceeded under mild conditions, enabling the introduction of sensitive functional groups, such as aldehydes and a fluorescent xanthone moiety. Importantly, the reaction exhibited good chemoselectivity, leaving reactive side chains in residues such as Ser, Tyr, and Arg untouched, although residues like His and Met, which are also potentially reactive, were not tested. The mildness of the thianthrenium salt synthesis, together with the efficiency of the C–H functionalization protocol, enabled its application to peptide‐drug conjugates preparation and peptide ligation strategies. Furthermore, the method was also compatible with cyclic peptides functionalization.

Exogenous directing groups can guide selective metalation of amino acid residues when installed either in amino or carboxyl groups, as well as in the side chain heteroatom, such as in Tyr or Trp. The installation of directing groups is, therefore, a powerful strategy to achieve selectivity in C–H functionalization of oligopeptides [122].

**SCHEME 30:**
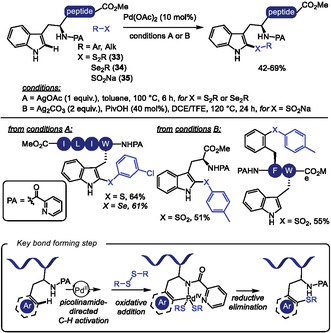
Pd‐catalyzed picolinamide‐directed C–H chalcogenation of aromatic amino acids.

Sharma and coworkers [125] exploited this strategy in the directed Pd(II)‐catalyzed C–H chalcogenation at the C2 position of N‐terminal Trp residues, using a picolinamide (PA) directing group (Scheme 30). In the presence of disulfides **33** or diselenides **34**, the reaction proceeds through a proposed mechanism involving initial picolinamide‐directed C–H activation by Pd(II), followed by oxidative addition to the dichalcogenide to generate a Pd(IV) intermediate, which underwent reductive elimination to form the desired product. The directing group could be efficiently removed under Zn/HCl conditions. The method tolerated dipeptides bearing hydrophobic amino acids; however, side chains with reactive functional groups required protecting group installation. In addition to aryl disulfides and diselenides, alkyl disulfides—including a cysteine‐derived reagent—also furnished the desired products, albeit with lower efficiency, thus overcoming previously reported limitations [126].

Shortly thereafter, Sharma's group further expanded the scope of Trp C2 chalcogenation by developing a Pd‐free protocol employing Ag(TFA) as an oxidant at room temperature [127]. This alternative approach enabled chalcogenation of C‐terminal and internal Trp residues within oligopeptides, offering a complementary and milder route for selective peptide modification.

A related picolinamide‐directed C–H activation strategy was also reported by Sharma and coworkers in 2024 for the sulfonylation of aromatic amino acids using sodium arylsulfinates **35** [128]. The reaction proceeds via a similar Pd(II)/Pd(IV) catalytic cycle, and mechanistic studies, particularly the use of TEMPO and BHT as radical scavengers, suggested the formation of a sulfonyl radical under the reaction conditions. This methodology was broadly applicable to Phe, Tyr, and Trp residues in di‐, tri‐, and tetrapeptides affording the corresponding sulfonylated products with comparable efficiency.

**SCHEME 31:**
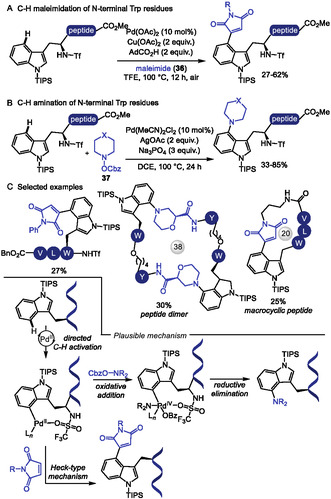
Tf‐directed Pd‐catalyzed C–H activation. (A) maleimidation; (B) amination; (C) selected examples of Tf‐directed C–H activation with maleimide or amine coupling partners. Tf = trifluoromethanesulfonamide.

The more challenging Pd‐catalyzed C4‐selective olefination of indoles has been achieved for the first time using a N‐terminal trifluoromethanesulfonamide (Tf) directing group [129]. After that, this strategy was applied to tryptophan residues in peptide macrocyclization [130]. In 2023, Liu and coworkers [131] reported the Pd(II)‐catalyzed Heck‐type C–H maleimidation at the C4 position of Trp residues embedded in small peptides (Scheme 31A). The reactions conditions were compatible with dipeptides containing Lys and Glu residues bearing protected amino and carboxyl groups. Additionally, peptides incorporating aromatic amino acids such as Phe and Tyr were well tolerated. Macrocyclic peptides were achieved from intramolecular reaction of di‐ and tripeptides bearing an appended maleimide unit **36**. Remarkably, the resulting macrocycles exhibit anti‐SARS‐CoV‐2 activity, binding to the viral nucleocapsid (N) protein and inhibiting viral replication.

Building on this strategy, Liu and Wang [132] extended the Tf‐directed Pd(II)‐catalyzed C–H activation to C4 amination of Trp, employing O‐benzoyl hydroxylamines **37** as electrophilic amine sources (Scheme 31B). DFT studies suggest that the aryl‐Pd(II) species, formed in the presence of the electron‐deficient Tf‐directing group, can activate the N–O bond of the amine source, leading to the formation of a Pd(IV) intermediate [133]. Benzoyl hydroxylamines derived from morpholine, piperazine, and pyrimidine were well tolerated under the reaction conditions, and conjugation of Trp with antidepressant paroxetine was achieved in 36% yield. However, other smaller‐ring cyclic, acyclic, and heteroaromatic amines failed to deliver the desired aminated products. The method also proved compatible with peptides bearing protected reactive functional groups such as Tyr, Thr, Asp, and Glu. Notably, morpholine‐derived unnatural *β*‐amino acid were used to construct peptide fragments, which were successfully coupled to Trp and Trp‐containing dipeptides. This highlights the synthetic utility of the method for assembling more complex peptide structures from smaller peptide fragments. Interestingly, when intramolecular cyclization was attempted, dimerization was observed, leading to the formation of stapled cyclodimeric peptides (Scheme 31C).

**SCHEME 32:**
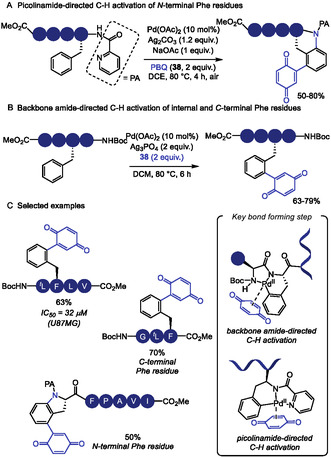
Pd‐catalyzed picolinamide‐directed C–H activation. (A) N‐terminal Phe residues; (B) internal and C‐terminal Phe residues; (C) selected examples of Phe functionalization.

The C–H functionalization of the aromatic ring in phenylalanine remains a significant challenge due to its inherently lower reactivity compared to other aromatic amino acids, such as tryptophan [20]. In 2023, Tang and coworkers [134] reported the installation of a picolinamide auxiliary in the amino group of Phe residue, which allowed for the Pd(II)‐catalyzed C–H activation for the modification of N‐terminal Phe residues in oligopeptides using *p*‐benzoquinone (**38**) as the coupling partner (Scheme 32A). This strategy enabled the incorporation of an indoline moiety into the peptide backbone through a tandem Heck‐type coupling and C–N cyclization sequence. Mechanistic studies, including the isolation of a benzoquinone‐functionalized intermediate, supported the pathway involving two successive ortho‐C–H activation steps. However, the transformation required the presence of an adjacent Pro residue, limiting the peptide scope.

To address this limitation, the same group later developed a complementary approach to benzoquinone incorporation into internal and C‐terminal of Phe residues (Scheme 32B) [135]. In this protocol, Pd(II) coordination to the peptide backbone amide nitrogen served as directing group for C–H activation, enabling broader peptide compatibility. In this case, however, C–N cyclization was not observed. The resulting benzoquinone‐containing peptides were evaluated against HeLa and glioma U87 MG cancer cell lines (Scheme 32C), demonstrating promising cytotoxic activity.

While C(sp^2^)‐H activation has been extensively studied, the functionalization of aliphatic C—H bonds remains significantly more challenging due to their inherently lower reactivity. In peptides, this challenge is further compounded by the presence of multiple chemically similar C(sp^3^)‐H bonds within the backbone, which complicates site‐selective activation [136]. One effective strategy involves coordination of palladium to backbone amide groups, enabling C(sp^3^)‐H functionalization at the N‐terminus of oligopeptides. However, such approaches have been frequently restricted to alanine residues, which limits the scope of peptides [22].

**SCHEME 33:**
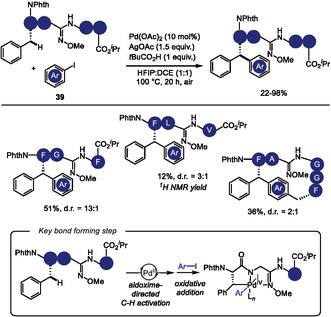
Pd‐catalyzed C(sp^3^)‐H arylation of internal and N‐terminal Phe residues.

To overcome these limitations, Hutton and coworkers [137] recently reported a strategy involving the incorporation of an aldoxime ether directing group into the peptide backbone to promote Pd‐catalyzed C(sp^3^)‐H arylation of both N‐terminal and internal phenylalanine residues (Scheme 33). The installation of the directing group requires a thioamide within the peptide sequence. Other amino acid residues such as leucine, alanine, and lysine can also be functionalized, if they were appropriately positioned relative to the directing group. When the aldoxime was positioned near sterically hindered residues, such as leucine and valine, both yields and diastereoselectivity decreased. Mechanistic insights supported by prior literature suggest a catalytic cycle involving initial C–H activation by a Pd(II) species followed by oxidative addition of aryl iodide **39** to form a Pd(IV)‐palladacycle intermediate. Intramolecular C–H activation furnished a macrocyclic pentapeptide. Importantly, the aldoxime ester could be removed under oxidative conditions, demonstrating the utility of the methodology in the modification of native peptides.

### 6.2 Rhodium

**SCHEME 34:**
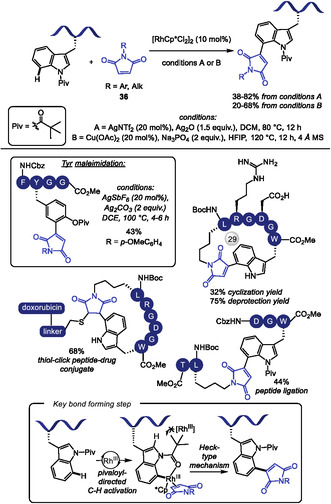
Rh‐catalyzed pivaloyl‐directed C–H maleimidation of Trp and Tyr residues.

Rh‐catalyzed regioselective C–H olefination of the challenging C7 position of tryptophan was accomplished for the first time with the assistance of a pivaloyl directing group that effectively blocks the more reactive C2 position [138]. Inspired by this work, Zhu [139] and Wang and Liu [140] independently applied this strategy to the maleimidation of tryptophan residues under distinct reaction conditions (Scheme 34). Despite employing the same rhodium catalyst precursor, the former used silver salts as both base and oxidant, whereas the latter employed Cu(OAc)_2_ as oxidant and Na_3_PO_4_ as base.

Both methodologies exhibited broad tolerance to a variety of maleimides **36** bearing different *N*‐substituents. Importantly, the maleimide moiety served as a versatile handle for conjugation of natural products, other amino acids and dipeptides, as well as fluorophores, at the C7 position of Trp. Selective C–H functionalization at Trp in di‐ and tripeptides containing phenylalanine was successfully achieved, though selectivity over other aromatic amino acids was not systematically investigated. Additionally, the methodologies proved applicable to short peptide macrocyclization and stapling strategies. A related Rh(III)‐catalyzed C–H activation protocol, using silver salts, was also reported for the site‐selective incorporation of benzoquinone **38** into dipeptides [141].

Zhu and coworkers further demonstrated the removal of both acid sensitive protecting groups and directing *N*‐pivaloyl group under trifluoroacetic acid conditions. They also showcased the utility of C7‐maleimide‐Trp in thiol‐click conjugation with biomolecules containing thiol groups and extended the strategy to rational design of a stapled RGD peptide‐drug conjugate incorporating doxorubicin. This conjugate exhibited enhanced selectivity, stronger binding affinity and superior cell penetrability compared to the free drug.

Similar conditions employing silver salts were applied to the Rh(III)‐catalyzed pivaloyl‐directed C–H maleimidation of tyrosine residues in oligopeptides comprising two to five amino acids, affording moderate yields (Scheme 34, example in detail, 38%–62%). As reported for tryptophan, macrocyclization was also achieved; however, succinimide rather than the expected Heck‐type product was formed, even under higher oxidant concentrations. This divergence is attributed to the conformational flexibility of succinimide ring, which likely suppresses *β*‐hydride elimination [142].

### 6.3 Iridium and Ruthenium

**SCHEME 35:**
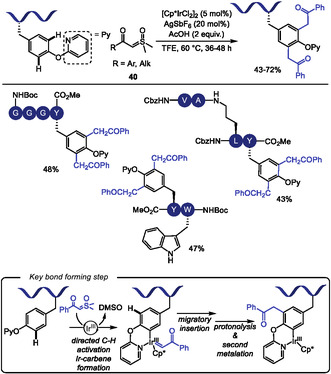
Ir‐catalyzed piridyloxy‐directed diacylmethylation of Tyr residues with sulfoxonium ylides.

Based on previously reported Pd‐ and Rh‐ catalyzed piridyloxy‐directed C–H olefination [142] and oxidations [143], Sakhuja and Bajaj [144] reported in 2024 an Ir‐catalyzed diacylmethylation of tyrosine residues in oligopeptides using sulfoxonium ylides **40** as a carbene precursor (Scheme 35). The C–H activation mechanism was supported by the isolation and mass spectrometric identification of iridacyclic intermediates. The reaction exhibited a broad scope with respect to ylide component, tolerating a variety of alkyl and (hetero)aryl groups bearing either electron‐donating or electron‐withdrawing substituents at the *para* or *meta* positions. Common *N*‐protecting groups such as Cbz, Ac, and Boc were well tolerated under the reaction conditions. While standard conditions allowed for the deprotection of N‐ and C‐terminal protecting groups, removal of O‐pyridyl directing group were not achieved. Although selectivity among different aromatic amino acids was not systematically examined, a difunctionalized tyrosine product was obtained in 47% yield from a dipeptide containing both tyrosine and tryptophan, suggesting a possible preference for directed over innate C–H activation under the reaction conditions.

**SCHEME 36:**
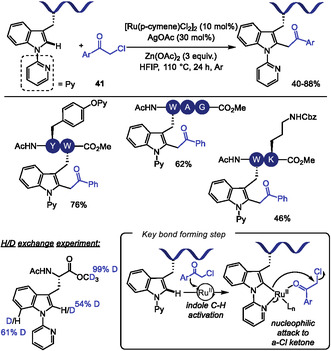
Ru‐catalyzed piridyloxy‐directed acylmethylation of Trp residues with *α*‐chloroketones.

Shorlty thereafter, Liu and Wang reported the Ru(II)‐catalyzed C–H acylmethylation of tryptophan using *α*‐chloroketones **41** as alkylation agents (Scheme 36) [145]. The method enabled the selective functionalization of di‐ and tripeptides in moderate to good yields (40%–85%). A broad range of *α*‐chloroketones, including those bearing electron‐withdrawing or electron‐donating substituents and heterocyclic moieties, were well tolerated. High selectivity for tryptophan alkylation was observed even in the presence of O‐Py‐protected tyrosine, affording the corresponding product in 76% yield. While gram‐scale functionalization and subsequent directing group removal were demonstrated for the amino acid, these transformations were not performed to the peptides. Notably, although C2‐alkylation was the sole product observed, H/D exchange experiments revealed that metalation at the C7 position also occurs, indicating a reversible C–H activation process. The formation of a five‐membered ruthenacycle at the more nucleophilic C2 position likely accounts for its higher reactivity toward acylmethylation compared to the six‐membered metallacycle at the less nucleophilic C7.

### 6.4 Copper and Iron

**SCHEME 37:**
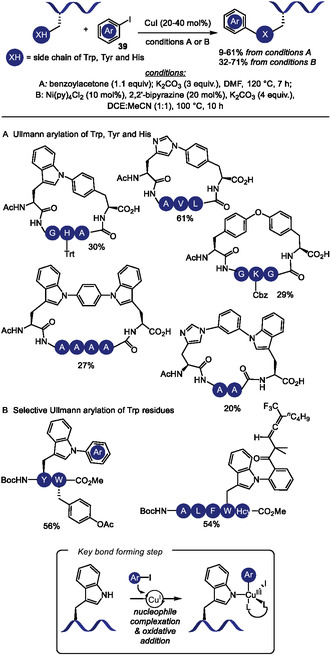
Ullmann‐type *N*‐arylation of aromatic amino acids in peptides.

Copper‐catalyzed reactions are reliable methods to construct C(sp^2^)‐N bonds in heterocyclic rings such as indole and imidazole [146, 147, 148], which can be found in Trp and His residues. In 2023, the copper‐catalyzed Ullmann‐type arylation of the heteroatom in the side chains of amino acid residues in native peptides has been developed as an effective strategy to promote cross‐linking, thereby modulating peptide backbone conformation (Scheme 37A) [149]. Under optimized conditions employing CuI/bezoylacetone catalytic system, macrocyclic products were obtained in moderate isolated yields (17%–61%) from (hetero)aromatic side chains such as those in Trp, His, and Tyr, and from primary amide group such as in Gln. Nonaromatic heteroatom containing amino acids, including Cys, Arg, and Lys, also furnished cyclized products, albeit in lower yields. In contrast, other heteroatom‐containing residues such as Ser, Thr, Met, and Glu, as well as internal backbone amides, showed no reactivity under the same conditions. In cases where multiple reactive sites are present within the peptide sequence, the use of appropriate protecting groups is required to achieve selective functionalization. When two suitable heteroatom‐containing amino acids are present in the peptide sequence, double coupling with external diiodoarene was possible, enabling the incorporation of diverse aromatic linkers of varying sizes, geometries, and electronic properties, thus offering potential for fine‐tuning peptide conformation. Notably, the reaction can also be performed on peptides immobilized on solid‐phase peptide synthesis (SPPS) resin, which minimizes the formation of side products and simplifies purification.

Using a different catalytic system based on Cu/Ni/2,2′‐bipyrazine, Yang and coworkers achieved Ullmann‐type arylation at tryptophan residues (Scheme 37B) [150]. Moderate to good yields (44%–78%) were obtained from the arylation of oligopeptides containing two to five amino acid residues. Notably, other potentially reactive amino acids, such as histidine and tyrosine, did not furnish the arylated product. This method was employed to incorporate trifluoromethyl allenyl tags, providing a handle for subsequent late‐stage peptide modifications.

**SCHEME 38:**
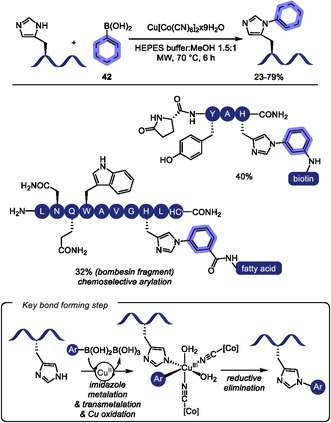
Heterogeneous Chan‐Lam arylation of His residues.

Despite the potential advantages, including easier handling, catalyst recovery, and enhanced stability, particularly under challenging biological conditions, heterogeneous catalysis has been neglected in the functionalization of biomolecules [151]. Recently, the use of heterogeneous copper catalysis has enabled the regioselective arylation and alkenylation of histidine residues in oligopeptide drug derivatives (Scheme 38) [152]. Employing copper(II) hexacyanocobaltate(III) as the catalyst and boronic acids **42** as arylating agents, various functional groups—including lipids, fluorescent labels, and reactive bioconjugation handles—were introduced at the His residue of short peptides in good yields (49%–79%) under aqueous conditions. In contrast, only modest conversions were observed for longer unprotected peptides (23%–56%). The method demonstrated high chemoselectivity, leaving nucleophilic side chains such as those of Tyr, Trp, Lys, and Arg, as well as backbone NH groups, unmodified, overcoming limitations reported in previous homogenous catalysis [153]. The selectivity between imidazole side chain and the backbone NH was attributed to steric hindrance at the internal amide positions. However, when a terminal amide precedes the His residue, arylation preferentially occurs ate the N‐terminus rather than the imidazole ring.

**SCHEME 39:**
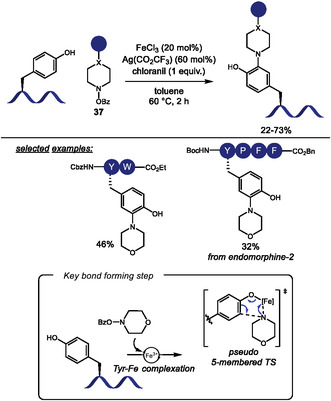
Fe‐catalyzed C–H aminoalkylation of Tyr residues.

C—N bond formation can also be achieved under iron oxidative catalysis [154, 155, 156]. In 2024, Yamada [157] reported the alkylamination of tyrosine in oligopeptides using O‐benzoyl‐hydroxylamines **37** as aminating reagent (Scheme 39). In contrast with the previously reported Fe‐catalyzed amination of phenols [91], which was unsuccessful in functionalizing tyrosine residues, the reaction was demonstrated not to proceed via a radical pathway. The mechanistic proposal instead involves the formation of highly electrophilic Fe(OCOCF_3_)_3_ in situ from silver trifluoroacetate and FeCl_3._ The iron–tyrosine complex formed then undergoes amination through an inner‐sphere electron transfer via a pseudo‐five‐membered cyclic transition state. This strategy enabled the selective alkylamination of tyrosine residues in di‐ and tripeptides containing other aromatic amino acids such as Trp and Phe. In addition, the functionalization of biologically important tetrapeptide endomorphin‐2 was also demonstrated. The method was used for the introduction of clickable handles, like azide and alkynes, a proteolysis labile linker for the synthesis of functional chimeric compounds, and for commercial drugs such as desloratadine. Although relying on chlorinated oxidants, silver salts, and chiral ligands, this method demonstrates its potential applicability as a tool for selective modification of biomolecules.

## 7 Free‐Radical Reactions

In recent years, free‐radical chemistry for the site‐selective modification of aromatic residues in biomacromolecules has advanced considerably, enabling the bioconjugation of peptides, proteins, and even tissues and living cells through chemical oxidation, photocatalysis, and electrochemistry. Chemical oxidation, although less applied, mainly promotes the formation of C—N and C—O bonds, allowing the selective incorporation of nitrogen heterocycles and the generation of stable DNA‐protein adducts for structural studies. Photocatalysis, more extensively studied, enables the construction of C—N, C—S, C—P, and C—C bonds under visible or near‐infrared light, supporting applications in cellular imaging, protein engineering, and therapeutic probe development. Electrochemistry favors O—S, C—S, and C—N bonds, facilitating virus and cell labeling, biosensors development, and the design of bioactive peptides.

### 7.1 Chemical Oxidation

**SCHEME 40:**
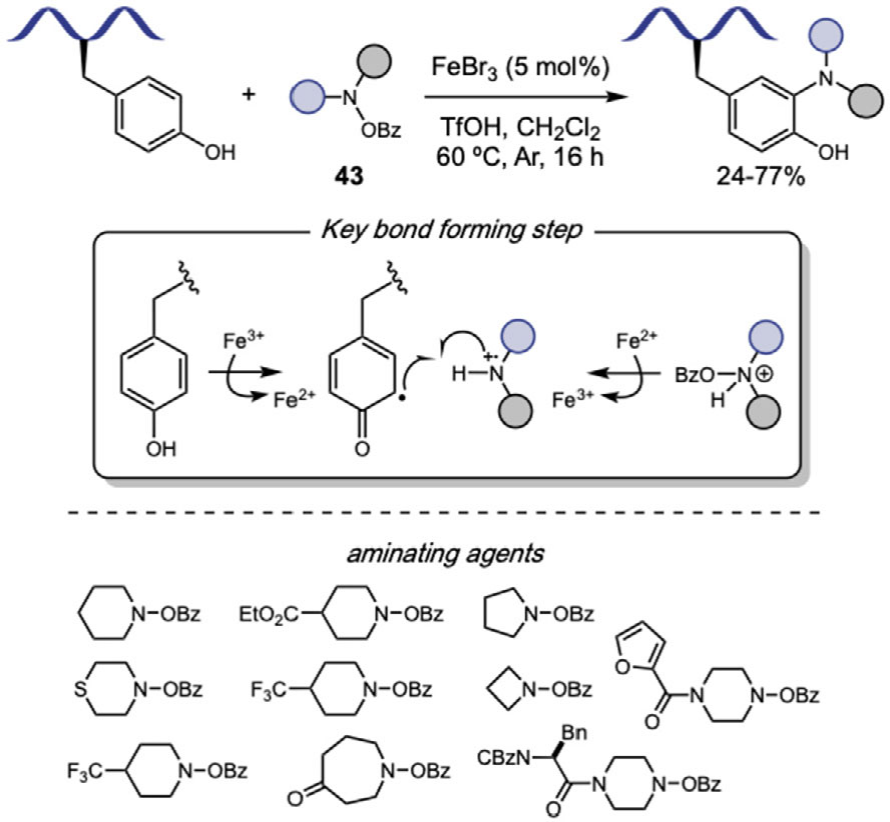
Late‐stage alkylamination of Tyr residues iron‐mediated.

Traditionally, C(sp^2^)–N bonds are constructed through indirect routes such as nitration/reduction or Buchwald–Hartwig couplings. In recent years, methods based on nitrogen‐centered radicals have enabled the direct amination of simple arenes. However, phenols remained challenging due to their redox‐active nature, with phenol ethers being more commonly used. Important precedents included the installation of phenothiazines in biomolecules [158], the amination of simple phenols via iron catalysis [159], and the use of Bronsted acids to stabilize aminyl radicals [160]. In 2024, a late‐stage functionalization strategy of tyrosine‐containing small peptides was developed using an iron‐mediated selective radical amination (Scheme 40) [161]. The method combined FeBr_3_ (5 mol%), TfOH (2.5 equiv.), and *N*‐(benzoyloxy)‐morpholine **43** as the aminating agent, achieving selective *ortho*‐amination relative to the phenol group and overcoming previously reported iron‐catalyzed amination protocol, which required for additional oxidants and chiral ligands [157].

The reaction proved efficient with Tyr‐containing peptides (di‐, tri‐, and tetrapeptides), tolerating sensitive residues such as Met, Cys, Thr, His, and Lys, and was applied even to bioactive peptides such as an endomorphin‐2 analog. In addition to morpholine, other heterocyclic amines, including piperidine, piperazine, and thiomorpholine, were successfully installed, albeit with lower yields. The methodology was also extended to a wide range of phenol‐based drugs, including estrone, estradiol, ibuprofen, neotame, paracetamol, mequinol, and even more complex structures such as amoxicillin and ezetimibe, in which case alternative catalyst conditions were required.

**SCHEME 41:**
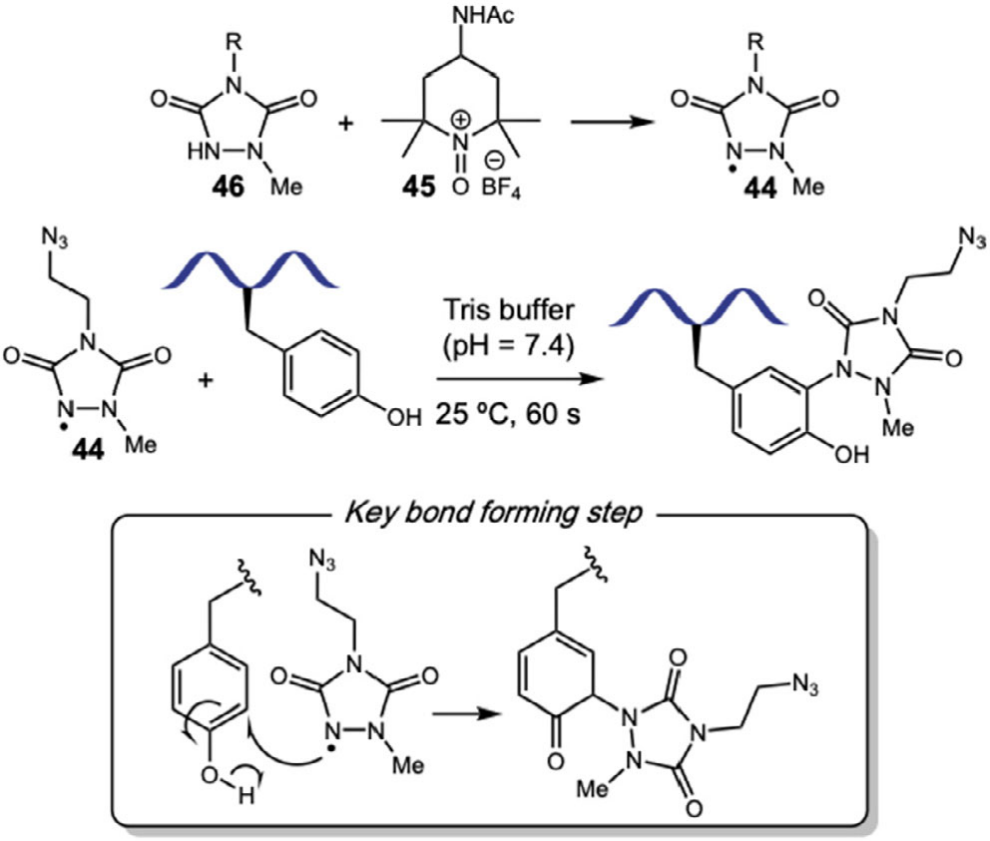
Tyr bioconjugation using an urazole radical.

Sato and coworkers reported a method for the selective bioconjugation of Tyr using stably generated *N*‐methylurazole radicals **44**, produced ex situ by oxidation with Bobbitt's salt (**45**) [162]. While traditional approaches based on N4‐substituted 1,2,4‐triazoline‐3,5‐diones (TADs, **46**) are limited by isocyanate formation and poor selectivity toward lysine and tryptophan [37], this radical‐based method offers a metal‐free, rapid, and Tyr‐selective alternative under physiological conditions (Scheme 41). The method proved effective with angiotensin II, complex proteins such as BSA and trastuzumab, and even enabled double modification at a single Tyr residue. Proteomic analyses in HEK293FT cells revealed more than 4,000 labeled Tyr sites, mainly located in solvent‐exposed and intrinsically disordered regions, highlighting the selectivity and broad application of the method.

### 7.2 Photocatalysis

**SCHEME 42:**
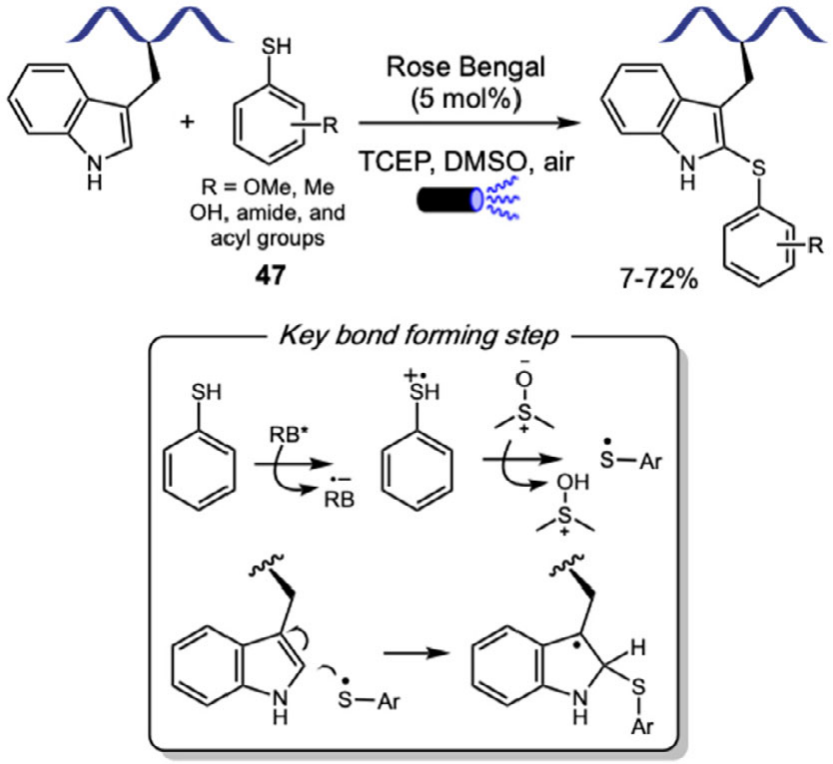
Photocatalyzed C–S bonding reaction.

In 2023, Sun and coworkers built on prior reports of sulfur‐centered radical generation from thiophenols under mildly oxidizing conditions [163] and developed the selective functionalization of bioactive peptides containing tryptophan through the formation of C—S bonds between the indole ring and thiophenol derivatives **47**, in a reaction mediated by visible light and DMSO (Scheme 42) [164]. Conducted under mild, open‐to‐air conditions, the method showed high selectivity for the C2 position of the indole and broad functional group tolerance as electron‐donating, electron‐withdrawing, and bioactive substituents, and was effective for both protected oligopeptides and complex bioactive peptides such as GLP‐1 and daptomycin.

**SCHEME 43:**
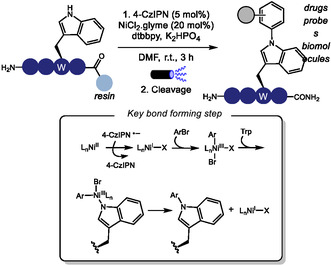
Solid‐phase metallaphotoredox arylation of Trp residues.

In the same year, recognizing the intrinsic challenge of indole nitrogen activation traditionally difficult with organometallic reagents due to the low nucleophilicity [165], Paixão and coworkers developed a selective metallaphotoredox methodology for the solid‐phase arylation of Trp residues, combining iridium and nickel catalytic cycles (Scheme 43) [166]. The method displayed a broad substrate scope, encompassing over 30 aryl halides, featuring heteroaryl groups and bioactive drug fragments, with yields ranging from 65% to 92%. The protocol proved effective for dipeptides, angiotensin II, oxytocin, GLP‐1, and leuprolide and enabled direct modification of resin‐bound peptides, demonstrating compatibility with solid‐phase peptide synthesis (SPPS).

**SCHEME 44:**
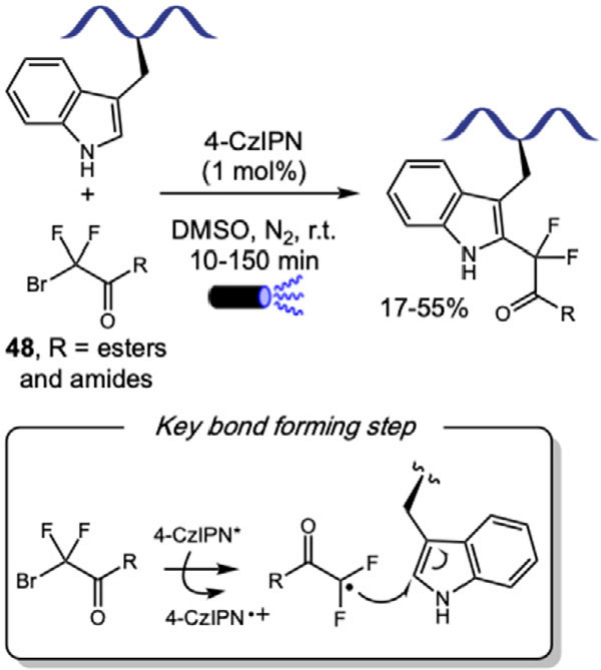
Photocatalytic Alkylation of Trp residues in peptides.

Inspired by the visible‐light‐induced difluoroalkylation of indoles previously reported [167], Mitchell and coworkers developed a photocatalytic method for the selective introduction of difluoroalkyl radicals, derived from bromodifluoroacetates and acetamides **48**, into tryptophan residues (Scheme 44) [168]. This rapid, additive‐free, visible‐light‐mediated reaction is operationally simple and was successfully applied to complex peptides, including Ac‐WHISKEY‐N_2_, DPP‐4 inhibitors, and ACE inhibitors, with typical yields ranging from 40% to 70%. A variety of functional handles such as PEG chains, biotin, alkynes, and sugars were successfully incorporated, and the reaction remained chemoselective in the presence of Cys, Lys, His, and Tyr, with no cross‐reactivity. Moreover, the incorporation of the difluoroalkyl group enables modulation of key physicochemical properties and allows precise reaction monitoring via ^19^F NMR.

**SCHEME 45:**
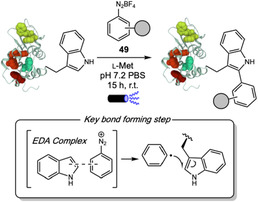
Photocatalytic arylation of Trp residues in peptides and proteins.

In 2024, our group introduced a platform for the late‐stage arylation of tryptophan residues in peptides and wild‐type proteins using aryldiazonium salts **49** under mild, biocompatible, metal‐free, and photocatalyst‐free conditions (Scheme 45) [169]. This advance contrasts with previous reports, which were limited to two examples of tripeptide arylation employing 20 mol% of Pd(OAc)_2_ [170]. The strategy demonstrates broad functional group tolerance, enabling the incorporation of chromophores, ^19^F NMR tag, azide, alkyne, and nucleophilic handles. Moreover, the method proved effective for the selective modification of complex biomolecules, including octreotride and lysozyme. These features underscore the potential of aryl group as versatile linkers for introducing complex functionalities, expanding the scope of bioconjugation strategies in chemically diverse biological environments.

**SCHEME 46:**
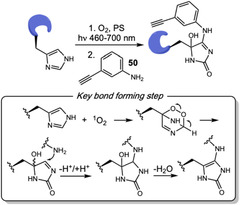
Labeling of histidine residues in living cells.

Recently, Li and collaborators extended the strategy for selective labeling of histidine residues through photocatalyzed singlet oxygen generation [171], making it suitable for application in living cells [172]. Histidine is an important amino acid in proteins but challenging to study due to its moderate reactivity and interference from more nucleophilic amino acids such as cysteine and lysine, which makes labeling selectivity difficult. In this work, singlet oxygen, generated from 17 different photosensitizers (e.g., eosin B, eosin Y, rose bengal, methylene blue, etc.) activated by visible light, was used to selectively oxidize histidine, making it more reactive toward nucleophilic chemical probes, such as 3‐ethynylaniline (**50**) (Scheme 46). Using this approach, approximately 7,200 unique histidine sites were identified across more than 2,400 proteins using single‐shot LC‐MS/MS. The technique enabled the discovery of histidine essentials for enzymatic activity, functional regions in metalloproteins, protein–protein interactions, and subcellular regulatory mechanisms, demonstrating its strong potential to uncover previously uncharacterized protein functions in both physiological and pathological contexts.

**SCHEME 47:**
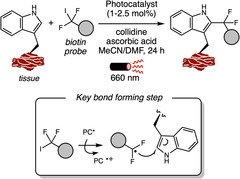
NIR photocatalysis for protein labeling in complex tissues.

Photocatalytic difluoroalkylation strategies typically rely on blue‐light irradiation, such as the method reported by Mitchell and coworkers [168], which may induce photodamage to proteins and biological tissues. To address this limitation, Fadeyi and coworkers developed an innovative approach based on near‐infrared (NIR) light‐activated photoredox catalysis (Scheme 47) [173]. Using porphyrin or helical carbenium ions as photocatalysts, they demonstrated the generation of aliphatic, aromatic, and heterocyclic difluoroalkyl radicals, as well as PEG chains, capable of selectively labeling Trp residues in bioactive peptides such as angiotensin II, oxytocin, DPP‐4, and ACE inhibitors, along with complex proteins including BSA and myosin. This mild, metal‐free protocol proved effective for protein modification in living cells and in normal and tumor human tissues, exhibiting deep NIR light penetration and preserved reactivity under physiological conditions. The method further enables applications in molecular imaging and proteomic analysis, underscoring its broad utility across complex biological systems.

**SCHEME 48:**
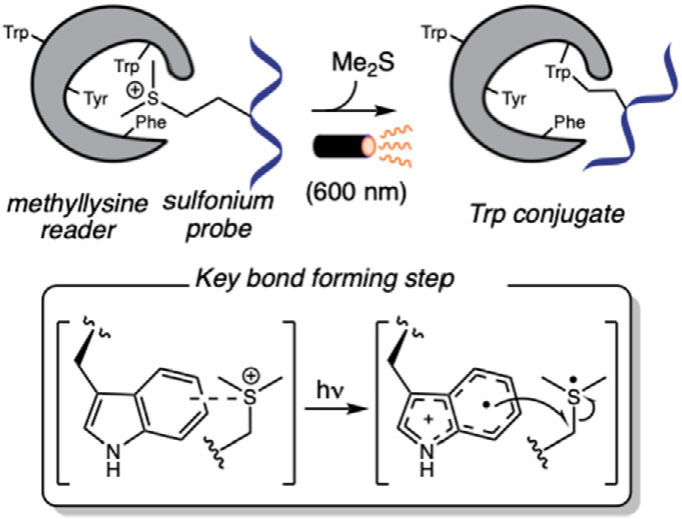
Photocrosslinking of methyllysine reader proteins.

In 2024, Feng and coworkers presented an original photocrosslinking strategy based on a UVB‐induced reaction between a peptide probe containing norleucine‐*ε*‐dimethylsulfonium and tryptophan residues within methyllysine reader proteins (Scheme 48) [174]. Unlike traditional azide or diazirine based methods, which often suffer from low selectivity due to the generation of reactive nitrene or carbene intermediates [175], this approach leverages a *σ*–*π* donor–acceptor interaction between the sulfonium group and the photoexcited tryptophan, enabling highly selective covalent labeling within aromatic cages.

This method successfully identified multiple methyllysine reader families, including CBX1, BPTF, JMJD2A, mORC1, and dSfmbt, without nonspecific labeling. The approach was extended to cellular lysates, where it enabled proteome‐wide identification of known readers, such as BPTF, PHF2, TAF3, CHD1, ING3, and the discovery of novel ones, such as BRWD3, validated as a new H3K4me3‐binding protein. Moreover, this Trp‐sulfonium photocrosslinking chemistry was successfully applied to other protein‐ligand systems involving S^+^–*π* interactions, including betaine/choline‐binding proteins and engineered peptide‐protein complexes.

In 2025, the authors extended this sulfonium‐based crosslinking methodology to target methylarginine reader, achieving selective crosslinking with both tryptophan and tyrosine residues, thereby expanding its applicability across diverse reader protein families [176].

**SCHEME 49:**
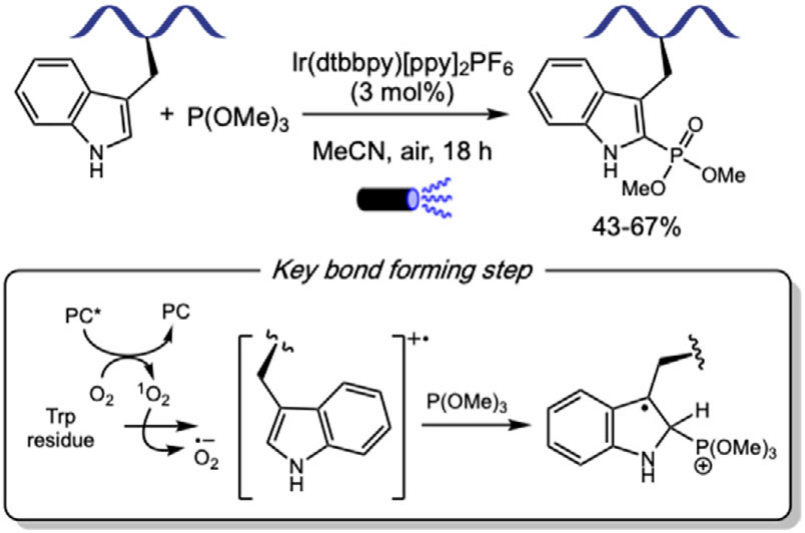
Visible‐light‐mediated phosphonylation of tryptophan residues.

Zhao and coworkers presented the first strategy for the phosphonylation of tryptophan residues under mild conditions visible‐light mediated (Scheme 49) [177]. The method successfully modified protected and unprotected tryptophans, di‐ and tripeptides containing Phe, Leu, Val, and Glu, as well as complex natural peptides such as endomorphin and segetalins A and B, with yields ranging from 48% to 65%. The method tolerates aliphatic and aromatic residues but is incompatible with unprotected OH(Tyr), NH_2_(Lys), and SH(Cys) functionalities due to oxidative side reactions. Importantly, phosphonylated segetalin derivatives exhibited enhanced antiproliferative activity against colon and liver cancer cell lines compared to their natural counterparts.

**SCHEME 50:**
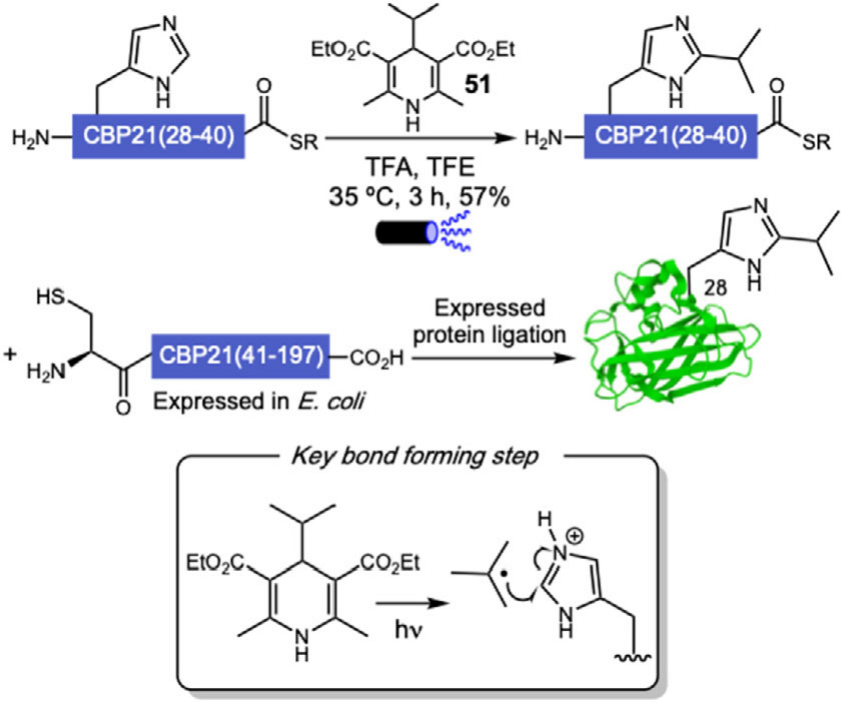
Visible‐light‐mediated His28 residue modification in CBP21.

Building on previous His‐selective peptide alkylation protocol [178], Ye and coworkers reported in 2025 a visible‐light‐mediated Minisci‐type C–H alkylation for the selective modification of histidine residues in proteins, using 4‐alkyl‐1,4‐dihydropyridines (DHP, **51**) as dual oxidants and radical precursors [179]. The method was applied to the chitin‐binding protein CBP21, targeting the C2 position of the His28 residue, which is essential for its catalytic activity. The methodology combined C–H alkylation with expressed protein ligation, enabling the semisynthesis of CBP21 variants bearing different groups on the histidine (Scheme 50). Protein integrity was validated by HPLC, MS, CD, and SDS‐PAGE, while functional assays revealed that these modifications reduced the chitinolytic activity of the enzyme, most likely by sterically hindering copper ion coordination.

### 7.3 Electrosynthesis

**SCHEME 51:**
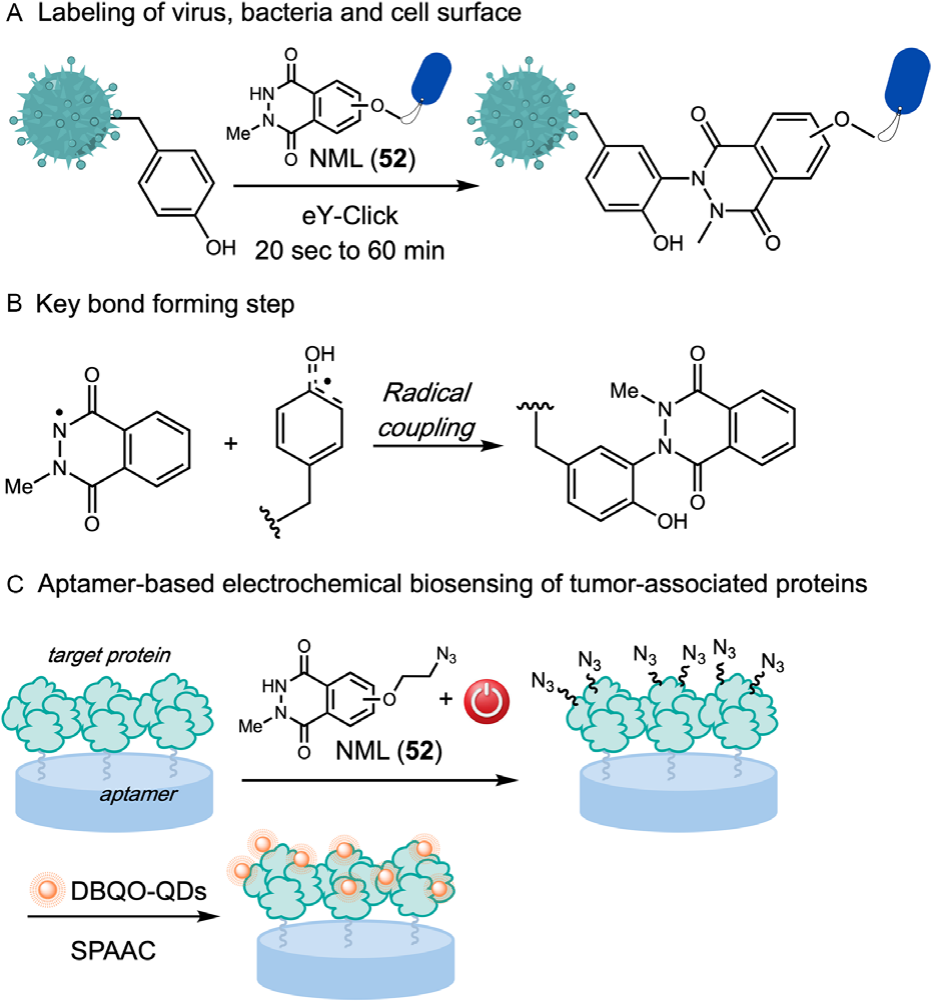
Click‐electrochemistry bioconjugation employing NML.

The development of selective methods for cell‐surface functionalization has relied on strategies such as metabolic oligosaccharide engineering and classical conjugations targeting lysine or cysteine residues. While powerful, these approaches present limitations, including long incubation times, dependence on cellular machinery, or restriction to highly nucleophilic amino acids. Recent advances in tyrosine‐selective chemistry have provided the basis for more direct approaches to surface modification [36, 180]. Building on these studies, Goin and coworkers applied mild electrochemical activation of *N‐*methylluminol derivatives (**52**) to rapidly and selectively modify exposed tyrosines on viral capsids, bacterial envelopes, and mammalian cell surfaces (Scheme 51A,B) [181]. Using this eY‐click, recombinant AAV2 vectors were decorated with GalNAc or mannose derivatives in seconds while maintaining structural integrity and infectivity, even achieving improved transduction in GalNAc‐receptor‐positive HuH‐7 cells. Gram‐negative and Gram‐positive bacteria were efficiently labeled at their membranes without affecting proliferation, and mammalian cells underwent rapid surface biotinylation with preserved viability.

Based in a similar eY‐click methodology, in 2025, an innovative aptamer‐based biosensing strategy was developed by Li and coworkers using a universal signal transduction module [182]. The method employs **52**, which is activated in situ under mild electric potential, enabling rapid and selective conjugation to tyrosine residues on proteins captured by aptamers immobilized on electrodes via epoxy‐silane chemistry. Subsequently, electroactive quantum dots functionalized with dibenzocyclo‐octyne‐amine (DBCO‐QDs) are attached through a SPAAC generating an amplified electrochemical signal (Scheme 51B,C). This approach, which avoids toxic catalysts and does not require specific structural features of the aptamer or target protein, demonstrated high sensitivity (limit of detection of 0.41 pg/mL) for tumor‐associated proteins such as MUC1, CEA, and AFP. Additionally, it is scalable for the detection of tumor cells, highlighting the importance of this bioconjugation strategy and its broad potential for clinical applications in cancer diagnostics.

**SCHEME 52:**
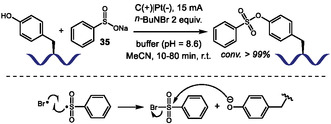
Electro‐induced O–S bonding reaction.

Building on advances in oxidative coupling and electrosynthesis [183], Weng and coworkers developed an efficient and selective electrobioconjugation method for tyrosine residues based on the anodic oxidation of aryl sulfinates **35** leading to the formation of sulfonated tyrosine derivatives under mild conditions (Scheme 52) [184]. This study demonstrated excellent site‐specificity, delivering remarkable selectivity over other aromatic residues, with broad applicability to both protected and unprotected peptides, bioactive natural peptides, and even proteins such as myoglobin, without causing degradation. Furthermore, benzenesulfonate‐labeled peptides exhibited antifungal activity, highlighting the potential of this method for therapeutic and industrial applications, particularly in protecting crops from agricultural pathogens.

**SCHEME 53:**
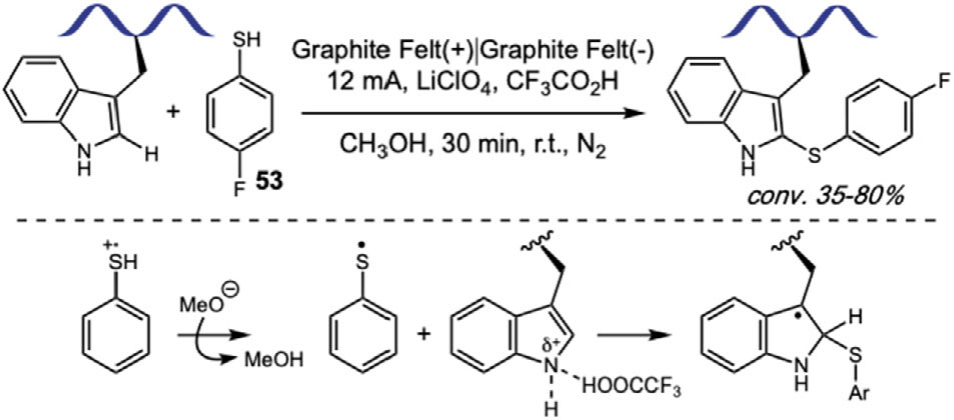
Electro‐induced C–S bonding reaction.

Building on the dehydrogenative C–H/S–H cross‐coupling of (hetero)aryl thiols with electron‐rich arenes [185], a selective electrochemical C—S bond formation strategy was developed for the labeling of tryptophan residues with thiophenols **53** (Scheme 53) [186]. The reaction employs a low‐cost graphite felt electrode and proceeds under mild conditions, free of metals, redox agents, and additives. This strategy demonstrated high selectivity and broad applicability for the functionalization of polypeptides containing a single Trp residue, including therapeutic and cyclic peptides such as leuprorelin, lanreotide, and eptifibatide, achieving conversions of 60%–90%. Moreover, the incorporation of fluorinated tryptophan enabled NMR‐based analytical applications, further expanding the utility and scope of this approach.

**SCHEME 54:**
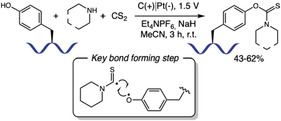
Electrochemical thiocarbamylation of Tyr‐containing peptides.

In the same year, Pan and coworkers reported a late‐stage electrochemical thiocarbamylation of tyrosine residues using an undivided cell setup. The reaction was carried out in acetonitrile with Et_4_NPF_6_ as the supporting electrolyte, at a constant potential of 1.5 V vs. Ag/AgCl, and in the presence of NaH (Scheme 54) [187]. This methodology enabled the functionalization of dipeptides and six polypeptides, affording the corresponding products in isolated yields ranging from 43% to 62%.

## 8 Summary and Outlook

In recent years, the bioconjugation of aromatic amino acids has advanced significantly, establishing itself as a versatile tool for chemical biology and drug discovery. Transition‐metal catalysis and radical‐mediated activation have become central strategies for late‐stage functionalization, with tryptophan and tyrosine emerging as the most frequently modified residues, while histidine and phenylalanine remain relatively underexplored.

Tryptophan continues to be the most versatile aromatic residue, displaying a wide range of electrophilic aromatic substitution, photocatalytic, and transition‐metal‐catalyzed reactions that enable selective formation of C—S, C—N, C—O, and C—C bonds under mild, biocompatible conditions. This reactive diversity, coupled with applicability in complex systems, has solidified tryptophan as a key target for bioconjugation strategies.

Tyrosine and phenylalanine exhibit complementary reactivity patterns. Tyrosine chemistry has evolved from classical electrophilic aromatic substitution to electrochemical and enzymatic transformations, enabling selective modification of proteins, antibodies, viruses, cells, and bacteria, thereby expanding its potential for biological applications. Phenylalanine, traditionally considered inert, has been successfully functionalized via palladium‐catalyzed C–H activation, although these reactions still require high temperatures and organic solvents, limiting their biocompatible applicability.

For histidine, selective modification has primarily been achieved through nitrogen‐based nucleophilic reactions under biocompatible conditions. More recently, photocatalytic methodologies have explored new reactivities of this residue under mild conditions, enabling bioconjugation in living cells and demonstrating the potential of in situ approaches to expand its chemical scope.

Despite significant advances in selectivity and scope, many methodologies still rely on optimized model peptides, and their application to more complex biological systems remains a central challenge. Nonetheless, the field of aromatic amino acid functionalization is rapidly expanding, transitioning from harsh chemical conditions to milder, more selective, and biocompatible approaches capable of preserving biomolecular structure and function. Overcoming current limitations, particularly regarding biological compatibility and the exploration of new residue reactivities, will be decisive for reconciling synthetic versatility with biological complexity, driving the next generation of site‐selective molecular engineering methodologies (Table 1).

**TABLE 1:** Summary.

Amino acid residue	Reaction type	Functionalization	Reaction conditions	Target	Ref
Histidine	Heteroatom as nucleophile	Aza‐Michael	Acrolein, PBS buffer (pH 7.4), 25°C, 30 min, labeled hydrazine	Proteins	[83]
Histidine	Heteroatom as nucleophile	*N*‐sulfonylation	EM12‐SO2Im, 24 h	Proteins	[87]
Histidine	Biocatalysis	Aza‐Michael	Ferritin‐based metalloenzyme, Tris buffer, 37°C, 12 h	Peptides and proteins	[100]
Histidine	Transition metal catalysis	*N*‐arylation	CuI, benzoylacetone, K_2_CO_3_, DMF, 120°C, 7 h	Peptides	[149]
Histidine	Transition metal catalysis	*N*‐arylation	Cu[Co(CN)_6_]_2_x·9H_2_O, HEPES buffer:MeOH (1.5:1), MW 70°C, 6 h	Peptides	[152]
Histidine	Photocatalysis	Oxidation/C—N bond formation	Photosensitizer, HBSS, 37°C, 1–11 h, 560 nm LED	Live cells	[172]
Histidine	Photocatalysis	Alkylation	DHP reagent, TFA and TFE, Ar, 35°C, 3 h, Blue LED	Peptides	[179]
Phenylalanine	Transition metal catalysis	C–C and C–N coupling	Pd(OAc)_2_, Ag_2_CO_3_, NaOAc, DCE, 80°C, 4 h, air	Peptides	[134]
Phenylalanine	Transition metal catalysis	Alkenylation	Pd(OAc)_2_, Ag_3_PO_4_, DCM, 80°C, 6 h	Peptides	[135]
Phenylalanine	Transition metal catalysis	Csp^3^–H arylation	Pd(OAc)_2_, AgOAc, PivOH, HFIP:DCE (1:1), 100°C, 20 h	Peptides	[137]
Tryptophan	EAS	C—S bond formation	*p*‐chloro‐trifluoromethanesulfenamide, BF_3_.OEt_2_ or TfOH, DCM or DCE, r.t. to 50°C, 24–48 h	Peptides	[29]
Tryptophan	EAS	C—S bond formation	Cys(Acm)(O), Gn.HCl, TFA, 25°C, 1 h (for Trp) or Cys(Acm)(O), TMSOTf, Gn.HOTf, TFA, 25°C, 1 h (for Tyr)	Peptides	[32]
Tryptophan	EAS	C—S bond formation	8‐quinoline thiosulfonate, TFA, 30°C, 1 h	Peptides	[33]
Tryptophan	EAS	C—N bond formation	Triazole, KI, KIO_3_, MeSO_3_H, HCO_2_H, DMSO/H_2_O, 0°C, 1 h	Peptides	[35]
Tryptophan	EAS	Alkylation	Thiophene‐ethanol derivative, In(OTf)_3_, ^*n*^Bu_4_NPF_6_, HFIP, r.t., 5–20 min	Peptides and proteins	[48]
Tryptophan	EAS	Alkylation	Thiophene‐ethanol derivative, imidazolium‐sulfonic acid catalyst, HFIP, r.t., 5 min	Cell lysates	[49]
Tryptophan	EAS	C—N bond formation	Triazine, TfOH, HFIP, r.t., 8–24 h	Peptides	[50]
Tryptophan	Addition	Petasis reaction	Glyoxylic acid monohydrate, 1,4‐dioxane, 80°C, 3–16 h	Peptides	[66]
Tryptophan	Addition	Oxidative cyclization	N_3_‐ABNOH, PBS buffer (pH 7.5), TEMPO^+^·BF_4_^−^, r.t., 1 h	Antibodies	[69]
Tryptophan	Addition	C—O—N bond formation	AcOH, NaNO_2_ (w/ or w/o), 1 h, 25°C or NaHCO_3_ buffer, 37°C, 18 h	DNA	[71]
Tryptophan	Addition	Oxidative cyclization	*N*‐sulfonyl oxaziridine, PBS, MeOH, 10 min	Peptides and proteins	[79]
Tryptophan	Addition	Oxidative addition	Diaryl nitrones, PBS buffer, CH_3_CN, 370 nm LED, 5 min	Peptides and proteins	[80]
Tryptophan	Heteroatom as nucleophile	*N‐*alkylation	MBHC, DABCO, DCM, r.t., 1–15 h	Peptides	[85]
Tryptophan	Biocatalysis	*N*_(in)_‐alkylation	Alkylpyrophosphates, MgCl_2_, AcyF, NaCl, HEPES (pH 7.5), TCEP, DMSO, 37°C, 72 h	Peptides	[98]
Tryptophan	Biocatalysis	C(6)‐prenylation	Alkyl diphosphates, IPT, Tris/CaCl_2_ (pH 8), 37°C, 16 h	Peptides	[99]
Tryptophan	Biocatalysis	C(6)‐bromination	Trp halogenase, O_2_, NaBr, cofactor recycling, PBS, 25°C	Peptides	[106]
Tryptophan	Biocatalysis	C(6)‐bromination	Trp halogenase, O_2_, NaBr, cofactor recycling, PBS, 25°C	Peptides and proteins	[107]
Tryptophan	Transition metal catalysis	C(2)‐arylation	Pd(OAc)_2_, 2‐propanol, 70°C, 16 h	Peptides	[123]
Tryptophan	Transition metal catalysis	C(2)‐chalcogenation	Pd(OAc)_2_, AgOAc, toluene, 100°C, 6 h	Peptides	[125]
Tryptophan	Transition metal catalysis	C(2)–S bond formation	Pd(OAc)_2_, Ag_2_CO_3_, PivOH, DCE/TFE, 120°C, 24 h	Peptides	[128]
Tryptophan	Transition metal catalysis	C(4)‐alkenylation	Pd(OAc)_2_, Cu(OAc)_2_, AdCO_2_H, TFE, 100°C, 12 h, air	Peptides	[131]
Tryptophan	Transition metal catalysis	C(4)–N bond formation	Pd(MeCN)_2_Cl_2_, AgOAc, Na_3_PO_4_, DCE, 100°C, 24 h	Peptides	[132]
Tryptophan	Transition metal catalysis	C(7)‐alkenylation	[RhCp*Cl_2_]_2_, AgNTf_2_, Ag_2_O, DCM, 80°C, 12 h [RhCp*Cl_2_]_2_, Cu(OAc)_2_, Na_3_PO_4_, HFIP, 120°C, 12 h	Peptides	[139, 140]
Tryptophan	Transition metal catalysis	Diacylmethylation	[Ru(p‐cymene)Cl_2_]_2_ AgOAc, Zn(OAc)_2_, HFIP, 110°C, 24 h	Peptides	[145]
Tryptophan	Transition metal catalysis	*N*‐arylation	CuI, benzoylacetone, K_2_CO_3_, DMF, 120°C, 7 h	Peptides	[149]
Tryptophan	Transition metal catalysis	*N*‐arylation	CuI, 2,2′‐bipyrazine, Ni(py)_4_Cl_2_, K_2_CO_3_, DCE:MeCN (1:1), 100°C, 10 h	Peptides	[150]
Tryptophan	Photocatalysis	C—S bond formation	Rose Bengal, TCEP, DMSO, air, Blue LED	Peptides	[164]
Tryptophan	Photocatalysis	*N*_(in)_‐Arylation	4‐CzIPN (5 mol%), NiCl_2_ glyme (20 mol%), dtbbpy (20 mol%), K_2_HPO_4_, DMF, 25°C, 3 h, Blue LED	Peptides	[166]
Tryptophan	Photocatalysis	C(2)‐Alkylation	4‐CzIPN (1 mol%), DMSO, N_2_, r.t., 10–150 min, Blue LED	Peptides	[168]
Tryptophan	Photocatalysis	C(2)‐Arylation	Diazonium salt, KH_2_PO_4_, r.t., 3–15 h, Blue LED	Peptides and proteins	[169]
Tryptophan	Photocatalysis	Fluoroalkylation	TTMAPP (2 mol%), collidine, ascorbic acid, MeCN/DMF or PBS, 660 nm irradiation, 24 h	Peptides, proteins, cells, and tissues	[173]
Tryptophan	Photocatalysis	C—S bond formation	Methyllisine reader protein, Sulfonium peptide, Tris buffer, r.t., 5–30 min, UV‐A or UV‐B irradiation	Proteins	[174]
Tryptophan	Photocatalysis	C—S bond formation	Methylarginine reader protein, Sulfonium peptide, buffer (pH 7.5), r.t., 15 min, UV irradiation (365 nm)	Proteins	[176]
Tryptophan	Photocatalysis	Phosphonylation	[Ir(ppy)_2_(dtbbpy)]PF_6_ (3 mol%), MeCN, air, 18 h, Blue LED	Peptides	[177]
Tryptophan	Electrosynthesis	Thiophenylation	12 mA vs Graphite Felt/Graphite Felt, LiClO_4_, CF_3_CO_2_H, CH_3_OH, r.t., N_2_, 30 min to 5 h	Peptides	[186]
Tyrosine	EAS	C—S bond formation	Cys(Acm)(O), Gn.HCl, TFA, 25°C, 1 h (for Trp) or Cys(Acm)(O), TMSOTf, Gn.HOTf, TFA, 25°C, 1 h (for Tyr)	Peptides	[32]
Tyrosine	EAS	C—N bond formation	PTAD reagent, PBS (pH 7.4), r.t., 30 min	Peptides, proteins and cell lysates	[39]
Tyrosine	EAS	C—N bond formation	blocked triazolinedione‐indole, PBS/MeCN (1:1) (pH 7.3), 40°C, 24–48 h	Peptides and proteins	[40]
Tyrosine	EAS	C—N bond formation	Urazole‐containing peptide, NCS, Py, DMF, 0°C, 30 min; then 100 mM PBS (pH 8), MeCN, r.t., 1 min	Peptides	[41]
Tyrosine	EAS	C—N bond formation	TBD‐DO3A, PBS (pH 8.2), r.t., 24 h	Peptide	[47]
Tyrosine	EAS	Alkylation	Amine, formaldehyde, DIPEA, HFIP, r.t., 2 h	Peptides	[52]
Tyrosine	EAS	Fluorination	Selectfluor, PBS (pH 7), NaCl, 35°C, 4 h	Protein	[53]
Tyrosine	EAS	Chlorination	Methionine tetrapeptide, NCS, CHCl_3_, 25°C, 1–6 h	Peptides	[60]
Tyrosine	Heteroatom as nucleophile	O‐triflation	Triflate‐imidazolone, CsF, DMSO, r.t., 5–180 min	Peptides	[81]
Tyrosine	Heteroatom as nucleophile	O‐sulfonylation	Aryl sulfonyl‐azole reagent	Proteins	[88]
Tyrosine	Heteroatom as nucleophile	*O‐*difluoroalkylation	3,3‐difluoroallyl sulfonium salt, CBS, DMSO, 37°C, 1 h	Peptides	[89]
Tyrosine	Biocatalysis	C—S bond formation	Tyrosinase, O_2_, Na_3_PO_4_, NaCl, pH 7, 20°C, 30 min	Proteins	[114]
Tyrosine	Biocatalysis	Michael addition	Silk fibroin, tyrosinase, PBS, 35°C, 1 h	Proteins	[115]
Tyrosine	Biocatalysis	2,3‐dihybenzodrofuran ring formation	Tyrosinase, phosphate buffer (pH 6.5), 4°C, 30 min, CH_3_CN, vynil ether, 456 nm LED	Antibodies	[116]
Tyrosine	Biocatalysis	C—N bond formation	Laccase, O_2_, Tris buffer (pH 6), 37°C, 60 min	Proteins	[119]
Tyrosine	Transition metal catalysis	Alkenylation	[RhCp*Cl_2_]_2_, AgSbF_6_, Ag_2_CO_3_, DCE, 100°C, 5 h	Peptides	[142]
Tyrosine	Transition metal catalysis	Diacylmethylation	[Cp*IrCl_2_]_2_, AgSbF_6_, AcOH, TFE, 60°C, 36–48 h	Peptides	[144]
Tyrosine	Transition metal catalysis	O‐arylation	CuI, benzoylacetone, K_2_CO_3_, DMF, 120°C, 7 h	Peptides	[149]
Tyrosine	Transition metal catalysis	Alkylamination	FeCl_3_, Ag(CO_2_CF_3_), chloranil, toluene, 60°C, 2 h	Peptides	[157]
Tyrosine	Free radical	C–H amination	FeBr_3_ (5 mol%), TfOH, CH_2_Cl_2_, 60°C, Ar, 16 h	Peptides	[161]
Tyrosine	Free radical	C—N bond formation	Bobbitt's salt, Tris buffer, DMF (6%), 25°C, 60 s	Proteins	[162]
Tyrosine	Electrosynthesis	C—N bond formation	Luminol derivative, 750 mV vs Ag/AgCl, PBS, r.t.	Viruses, living bacteria and cells	[181]
Tyrosine	Electrosynthesis	C—N bond formation	ITO electrode functionalized with the aptamer (protein), PTDA‐N_3_, 750 mV vs Ag/AgCl, PBS, r.t.	Proteins	[182]
Tyrosine	Electrosynthesis	O—S bond formation	8–15 mA vs C/Pt, Na_2_HPO_4_ buffer pH = 8.6, ^*n*^Bu_4_NBr, MeCN, 25°C, 10–80 min	Peptides and proteins	[184]
Tyrosine	Electrosynthesis	Thiocarbamylation	1.5 V vs Ag/AgCl, Et_4_NPF_6_, NaOCH_3_, CH_3_CN, r.t., 3 h	Peptides	[187]

## Funding

This work was supported by Fundação Carlos Chagas Filho de Amparo à Pesquisa do Estado do Rio de Janeiro (E‐26/210.020/2024).

## Conflicts of Interest

The authors declare no conflicts of interest.

## Data Availability

The data that support the findings of this study are available from the corresponding author upon reasonable request.
